# Assessing the reproductive lifespan of female cancer survivors using AMH: a meta-synthesis of longitudinal studies

**DOI:** 10.1093/humupd/dmag019

**Published:** 2026-07-24

**Authors:** Neerujah Balachandren, Stavroula L Kastora, Richard A Anderson, Clara Luehwink, Melanie C Davies, Stuart A Lavery, Dimitrios Mavrelos, Ephia Yasmin

**Affiliations:** Reproductive Medicine Unit, University College London Hospital, London, UK; Reproductive Medicine Unit, University College London Hospital, London, UK; Centre for Reproductive Health, Institute for Regeneration and Repair, University of Edinburgh, Edinburgh, UK; Elizabeth Garrett Anderson Institute for Women’s Health, University College London, London, UK; Reproductive Medicine Unit, University College London Hospital, London, UK; Reproductive Medicine Unit, University College London Hospital, London, UK; Reproductive Medicine Unit, University College London Hospital, London, UK; Reproductive Medicine Unit, University College London Hospital, London, UK; Reproductive Medicine Unit, University College London Hospital, London, UK

**Keywords:** anti-Müllerian hormone, AMH, fertility, cancer, gonadotoxicity, ovarian reserve, ovarian insufficiency, chemotherapy

## Abstract

**BACKGROUND:**

Anti-Müllerian hormone (AMH) is widely used to assess gonadotoxicity related to anti-cancer treatments and to measure ovarian function in cancer survivors. The extent of AMH decline across malignancies and treatment regimens and the degree of subsequent recovery have not been systematically quantified.

**OBJECTIVE AND RATIONALE:**

The primary objective of this review was to quantify the change in AMH following cancer treatment and to model longitudinal AMH trajectories in survivors. The secondary objective was to compare AMH trajectories in cancer survivors with those of age-matched non-cancer controls. Data on long-term ovarian reserve after cancer treatment remain limited. This study integrates recent evidence and age-matched control data to better distinguish treatment-related AMH decline from normal reproductive ageing.

**SEARCH METHODS:**

A systematic search was performed across CENTRAL, MEDLINE, Google Scholar, Embase, and MedRxiv from inception until 24 January 2024, with an updated search on 19 March 2026. Both MEDLINE and Embase were searched via the Ovid platform, whilst Emtree index terms were used for EMBASE. The search strategy combined both index terms and free-text terms: (gonadotoxicity OR premature menopause OR premature ovarian insufficiency (POI) OR premature ovarian failure (POF)) AND (AMH OR Antimullerian* OR Anti-Mullerian) AND cancer. Studies were included if they involved female cancer patients of any age, including paediatric, adolescent and young adult (AYA), and adult populations, who received chemotherapy and who reported either pre- and post-treatment AMH levels or AMH measurements at defined time points after treatment. Studies were excluded if they did not report quantitative AMH data, involved male patients or non-human subjects, or included studies unrelated to cancer treatment-induced gonadotoxicity. The primary outcome was percentage change in AMH from baseline to 12 and 24 months from the end of treatment. Secondary outcomes included long-term AMH trajectories in adult and paediatric cancer survivors compared to the age-related decline in healthy controls. Risk of bias was assessed using the Joanna Briggs Institute (JBI) study-design-specific critical appraisal tools, and certainty of evidence was evaluated using GRADE. Analyses were performed in GraphPad Prism v10.6.

**OUTCOMES:**

Across all four cohorts (breast, lymphoma, thyroid, and paediatric/AYA mixed cancers), AMH levels were higher at 12 and 24 months compared with 6 months from the end of treatment. With breast cancer (median age 37.8 years, n = 2259), AMH was reduced by 76.4% at 12 months (95% CI: −93.5 to −55.7; 24 studies; GRADE: moderate-certainty evidence). With lymphoma, ABVD treatment regimens (median age 24 years, n = 146) were associated with a smaller reduction in AMH at 12 months (−21.1%; 95% CI: −81.6 to +14.8; four studies; GRADE: low-certainty evidence), whereas non-ABVD regimens (median age 26.3 years, n = 188) were associated with a larger reduction (−90.1%; 95% CI: −96.1 to −68.3; six studies; GRADE: moderate-certainty evidence). With thyroid cancer (median age 32.5 years, n = 181), radioactive iodine treatment was associated with a 57.9% reduction in AMH at 12 months (95% CI: −65.2 to −36.4; 4 studies; GRADE: high-certainty evidence). In the paediatric and AYA mixed cancers cohort (median age 9, n = 176), AMH was reduced by 63.1% at 12 months (95% CI: 64.3–61.9; four studies; GRADE: very low-certainty evidence). Among the adult and paediatric survivors who retained ovarian function, AMH trajectories remained relatively stable and closely aligned with age-matched healthy controls.

**WIDER IMPLICATIONS:**

AMH levels consistently improved between 6 and 12–24 months from the end of cancer treatment. We provide generalizable estimates of AMH trajectories in female survivors of both childhood and young adult cancer, indicating that their long-term AMH declines are similar to those of age-matched controls.

**REGISTRATION NUMBER:**

PROSPERO registration number: CRD420251000939.

## Graphical abstract

**Graphical Abstract:**
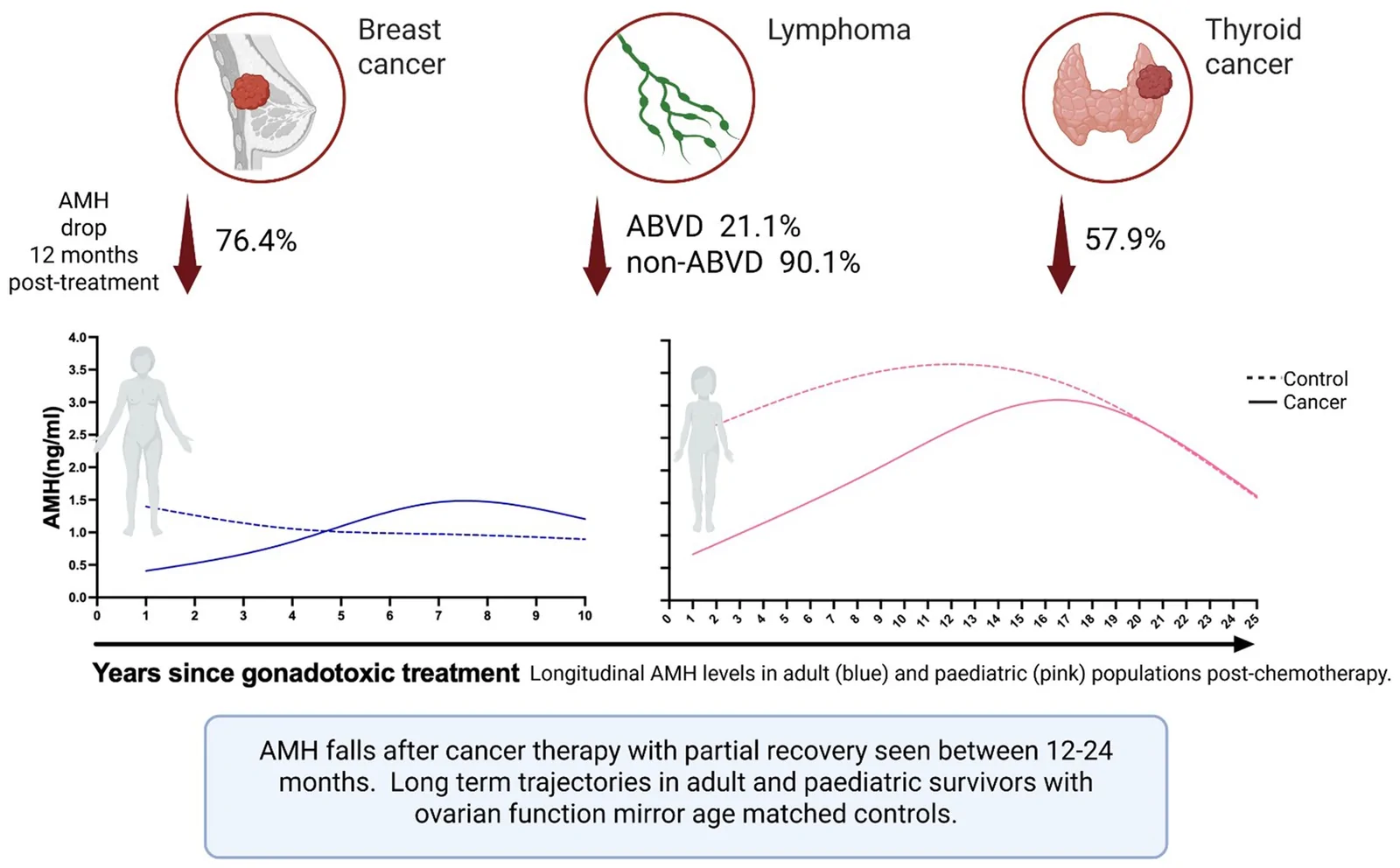
**AMH declines after cancer therapy, with partial recovery seen between 12 and 24 months.** Long-term trajectories of ovarian function in adult and paediatric survivors mirror those of age-matched controls. ABVD: treatment regimen including adriamycin, bleomycin, vinblastine, and dacarbazine. AMH, anti-Müllerian hormone.

## Introduction

Gonadotoxic anticancer treatments can profoundly impact the ovary by depleting both growing and primordial follicles (Morgan *et al.*, 2012). This follicular depletion may shorten the reproductive lifespan and in some women results in premature ovarian insufficiency (POI), with far-reaching consequences for fertility, endocrine health, and overall well-being (Panay *et al.*, 2024). The extent of ovarian damage depends on several factors, including the type, dosage, and duration of treatment, as well as the patient’s age and baseline ovarian reserve (Anderson and Cameron, 2011; Thomas-Teinturier *et al.*, 2013; Zavos and Valachis, 2016; Lambertini *et al.*, 2020).

Anti-Müllerian hormone (AMH) is increasingly utilized to assess the degree of gonadotoxicity related to anti-cancer treatments and to measure ovarian function in post-treatment cancer survivors (Anderson *et al.*, 2012; Anderson and Su, 2020; Su *et al.*, 2020; Cui *et al.*, 2023; Rotz *et al.*, 2025; Yano Maher *et al.*, 2025). Studies have demonstrated that gonadotoxic chemotherapy leads to a marked decline in AMH levels, with the degree of reduction correlating with the gonadotoxic potential of the treatment administered. This decline is observed across various age groups, including prepubertal girls, young adults, and older premenopausal women, underscoring the broad impact of chemotherapy on ovarian function (Anderson *et al.*, 2022).

The most commonly reported measure of ovarian toxicity after cancer treatment is the development of amenorrhoea. The risk of chemotherapy-induced amenorrhea (CIA) varies widely, ranging from 30 to 76%, depending on patient-specific factors and the chemotherapeutic agents used. Alkylating agents are particularly toxic to ovarian tissue, leading to higher rates of CIA (Morgan *et al.*, 2012). Highly intensive regimens, such as high-dose chemotherapy, conditioning prior to haematopoietic stem cell transplant, and total body irradiation (TBI), result in irreversible ovarian failure in over 90% of patients (Anderson and Wallace, 2011; Loren *et al.*, 2013).

Radiotherapy, especially when directed at the pelvis or administered as TBI, carries a high risk to ovarian function. A dose as low as 2 Gy has been shown to deplete ∼50% of the primordial follicle pool, while exposure to 20 Gy is associated with a near-certain risk of permanent ovarian ablation (Wallace *et al.*, 2003). Radiotherapy that includes the uterus additionally increases the risk of pregnancy complications (van der Kooi *et al.*, 2019; Rozen *et al.*, 2020).

Given these risks, fertility preservation strategies, such as embryo, oocyte, and ovarian tissue cryopreservation, have become integral to the management of young cancer patients (Anderson *et al.*, 2020; Mulder *et al.*, 2021; Su *et al.*, 2020). Additionally, the use of GnRH agonists (GnRHa) during chemotherapy has been explored as a means to preserve ovarian function, particularly in women with breast cancer (Lambertini *et al.*, 2018b). However, data on their effectiveness in preserving fertility and preventing early menopause are still evolving.

Despite these available options, a key challenge lies in accurately predicting the individual risk of ovarian insufficiency and tailoring fertility preservation strategies accordingly. While the gonadotoxicity of certain agents, particularly alkylating agents and platinum-based compounds, is well established, quantifying individual risk remains difficult. This becomes especially relevant when clinicians must weigh the potential risks and benefits of undertaking fertility preservation, particularly in urgent treatment settings, in patients with reduced medical fitness, or when the likelihood of the recovery of ovarian function is uncertain.

In the post-treatment setting, patients who resume menses after chemotherapy may falsely assume their fertility is intact, yet they remain at elevated risk of POI (Decanter *et al.*, 2010). Conversely, the consequences of a low post-treatment AMH measurement may be subject to over-interpretation. There is currently limited evidence on whether fertility preservation should be recommended before cancer treatment and which patients should be considered for it. The clinical value of monitoring ovarian reserve over time also remains uncertain. Long-term studies show that AMH recovery after chemotherapy is highly variable between individuals, with trajectories influenced by age at treatment, baseline ovarian reserve, and treatment type, making individual predictions challenging (Cameron *et al.*, 2019; Su *et al.*, 2020).

This study aims to quantify AMH decline following chemotherapy across various cancer types. Additionally, we aimed to estimate the long-term trajectory of AMH levels in female cancer survivors to better understand the lasting impact of treatment on ovarian function and reproductive potential. By improving our understanding of chemotherapy-induced ovarian toxicity and refining predictive tools, these findings may support more judicious use of fertility preservation strategies and support informed decision-making for patients facing cancer treatment.

## Methods

### Search strategy and selection criteria

A comprehensive systematic review was conducted according to a prospectively registered protocol (PROSPERO: CRD420251000939) and is reported in accordance with the Preferred Reporting Items for Systematic Reviews and Meta-Analyses (PRISMA) guidelines (Page *et al.*, 2021). Although the registered protocol specified inclusion of women of reproductive age (18–45 years), studies including paediatric and AYA populations were also included following screening, as they provided relevant longitudinal AMH data and contributed to understanding long-term reproductive trajectories after cancer treatment. This deviation from the protocol was made to ensure comprehensive capture of available evidence and is reported transparently.

A systematic search was performed across four electronic databases (Cochrane Central Register of Controlled Trials (CENTRAL), MEDLINE, Google Scholar, and Embase) and one preprint repository (MedRxiv), from inception until 24 January 2024, with an updated search on 19 March 2026. The search strategy combined both index terms (MeSH for MEDLINE; Emtree for Embase) and free-text terms. The following search terms were employed: ((gonadotoxicity OR premature menopause OR premature ovarian insufficiency (POI) OR premature ovarian failure (POF)) AND (AMH OR Antimullerian* OR Anti-Mullerian)) AND cancer (Supplementary Data File S1). Search terms were adapted per database screened.

### Data extraction

Following the removal of duplicates, two independent reviewers (N.B. and S.L.K.) screened the titles and abstracts of identified studies. Full-text articles deemed potentially eligible were retrieved and assessed for inclusion. Discrepancies between reviewers were resolved through discussion or consultation with a third reviewer (E.Y.). Data extraction was conducted independently by N.B. and cross-verified by S.L.K. to ensure accuracy and consistency.

The following information was systematically extracted using a standardized data extraction form.

Study characteristics included first author, publication year, country of origin, study design, and sample size (post dropouts).

Participant characteristics included age distribution (median and interquartile range (IQR)/mean and SD), baseline ovarian reserve indicators (e.g. AMH in ng/ml or pmol/l, AMH assay), and number of patients per cohort. For data harmonization, age distribution was converted to median (≈Mean) and IQR (Q1/Q3 ≈ Median ± 0.674 × SD). Additionally, AMH levels were converted when needed to ng/ml through the formula: ng/ml = (pmol/l)/7.14, assuming the molecular weight of AMH is 140 kDa.

Intervention details included type of malignancy, specific chemotherapeutic agents used, use of radioactive iodine (RAI), and any pelvic radiotherapy (or TBI).

Outcome measures included pre-chemotherapy AMH levels, post-chemotherapy AMH levels at specified time points in months/years from the end of treatment, and timing of AMH measurements relative to chemotherapy cycles.

Additional variables included follow-up duration reported in months or years.

### Inclusion criteria

Studies were considered eligible for inclusion if they included the following characteristics.

Population was female cancer patients who underwent chemotherapy/RAI. Studies involving adult, paediatric, and adolescent populations were included.

Intervention was administration of chemotherapeutic and/or radiotherapeutic agents. Outcomes were both pre- and post-chemotherapy AMH levels or random post-chemotherapy AMH levels, and timing of AMH measurements from diagnosis or end of gonadotoxic treatment.

Study design was original research, comprising randomized controlled trials, cohort studies, and case–control studies, published in peer-reviewed journals. Studies were excluded if they lacked quantitative data on AMH measurements before and/or after chemotherapy/radiotherapy/RAI or involved male patients or non-human subjects, or exclusively focused on populations with conditions unrelated to cancer treatment-induced gonadotoxicity. Case reports, editorials, conference abstracts without full manuscripts, non-peer-reviewed articles, or published in languages other than English without available translations were also excluded. Systematic reviews were not included in the analysis but were screened to identify additional eligible primary studies through reference list checking.

### Outcomes

The primary outcome of interest was the percentage change in AMH levels from baseline to post-chemotherapy at 12 and 24 months from the end of treatment. Secondary outcomes included AMH changes till end of follow-up per study in adult and paediatric cancer survivors versus the expected age-related changes in AMH in healthy controls.

### Quality assessment

Risk of bias was assessed independently by two reviewers (N.B., S.L.K.) using study-design-specific critical appraisal tools (Supplementary Table S1 and Supplementary Fig. S1) (Sterne *et al.*, 2016). Cohort studies were appraised using the Joanna Briggs Institute (JBI) Critical Appraisal Checklist for Cohort Studies, and uncontrolled longitudinal/case-series studies using the JBI Critical Appraisal Checklist for Case Series. Any disagreements between reviewers were resolved through discussion or consultation with a third reviewer (E.Y.). Certainty of the body of evidence for the prespecified primary outcomes was assessed separately using the GRADE framework (Guyatt *et al.*, 2008 and Schünemann *et al.*, 2013).

### Data analysis and quantitative synthesis

Data synthesis was performed using GraphPad Prism version 10.6 (Academic License). Descriptive statistics were calculated for each malignancy cohort, including median, and 95% CI: of AMH percentage change, i.e. (AMH post-AMH pre/AMH pre)×100, at 12 and 24 months. Continuous variables were summarized using medians and IQRs. Categorical variables were presented as frequencies and percentages. For studies that did not provide specific means/medians of pre- and post-chemotherapy AMH levels but provided graphical data, GraphReader and PlotDigitizer were used to extract numerical values to minimize data loss. For longitudinal data, five points were marked within each *x*-axis data point. Each data point (AMH at a given month/year of measurement) was measured three times to ensure accuracy, with the average value of the three points was used for analysis. Crude data per figure are presented in Supplementary Table S2. Studies reporting AMH levels from time of diagnosis, instead of the end of treatment, have been normalized by the addition of 6 months to the reported time scale. To describe the biphasic AMH trajectory, we fitted a segmented (piecewise) linear regression model with prespecified knots at 6 and 12 months from the end of treatment and estimated slopes before and after the knots.

An exploratory external-reference analysis was undertaken for studies reporting more than two post-treatment AMH time points without baseline data. Adult survivor trajectories were compared with published reference AMH data from non-cancer populations, and paediatric survivor trajectories were compared with published paediatric reference AMH data. To align age and time, the reported age at diagnosis and the interval from diagnosis or the end of treatment to AMH measurement were used to approximate age at each assessment. Because these comparator data were external to the included studies, this analysis was interpreted as exploratory and not as a formal comparative meta-analysis. Studies reporting only post-treatment AMH for more than two timepoints in either adult or paediatric populations were included in the longitudinal analysis. Studies reporting AMH post-treatment in mixed age populations (adults and young adolescent or paediatric cohorts) were reviewed separately and included in the systematic review (Supplementary Table S3).

## Results

### Study characteristics

A total of 820 studies were initially identified through systematic database searching. Following the removal of duplicates, 664 studies remained for title and abstract screening. After applying the predefined inclusion and exclusion criteria, 105 studies were selected for full-text review. Of these, 79 met eligibility criteria and were included in the qualitative analysis (Supplementary Data File S2), while 72 studies provided extractable data and were included in the quantitative synthesis (Fig. 1). The included studies encompassed a diverse range of malignancies, with varying chemotherapy regimens and follow-up durations.

**Figure 1.**
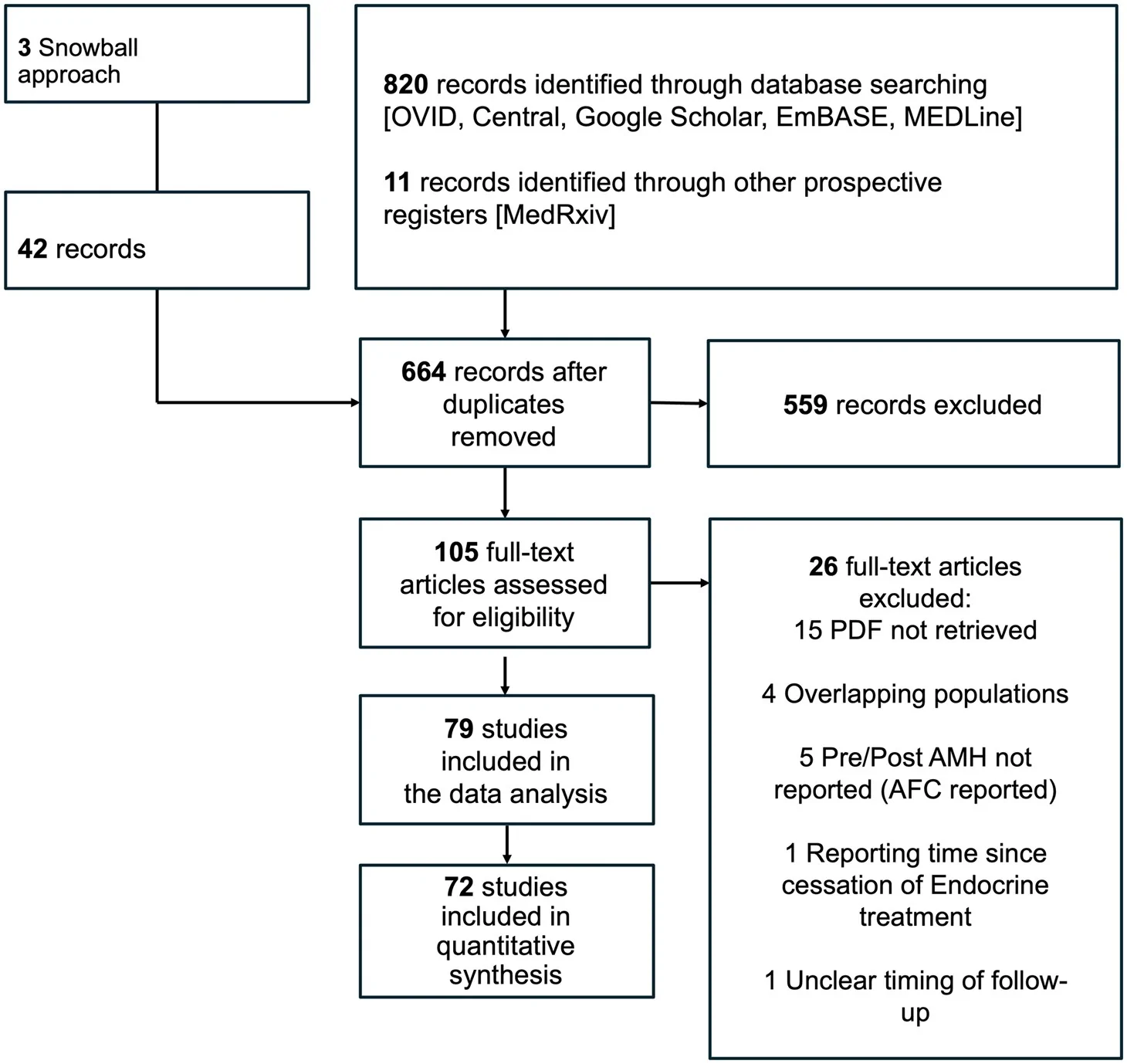
**PRISMA flowchart.** AMH, anti-Mullerian hormone.

Included studies were categorized by malignancy type and by follow-up approach: either those reporting both pre- and post-treatment AMH levels or those providing only longitudinal post-treatment AMH measurements without baseline data.

The baseline characteristics of included patients varied in terms of age at diagnosis, type of malignancy, and pre-treatment ovarian reserve, as reflected in their AMH levels (Table 1). Most studies reported AMH measurements at multiple post-treatment time points, with the most frequent being 6, 12, and 24 months from the end of treatment. For each malignancy type and cohort, the median change in AMH from pre-treatment to 12 and 24 months post-treatment was calculated (Table 1; Figs 2, 3, 4, and 5). Across all four cohorts (breast, lymphoma, thyroid, and paediatric/AYA mixed cancers), AMH levels were higher at 12 and 24 months compared with 6 months from the end of treatment (Table 1; Figs 2, 3, 4, and 5).

**Figure 2.**
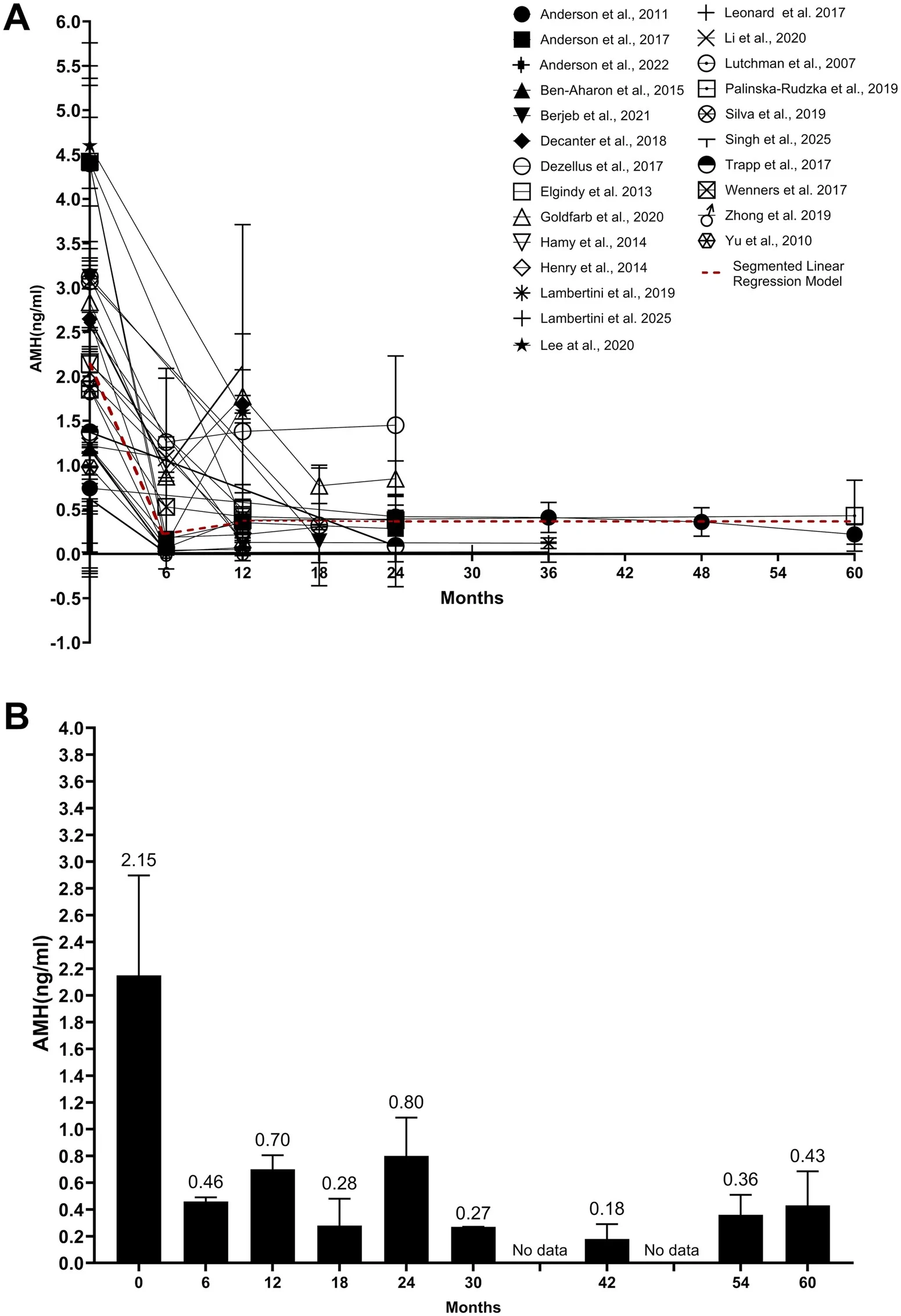
**Anti-Müllerian hormone (AMH) decline following chemotherapy in breast cancer patients.** (**A**) Data represent median and mean (SD) AMH values (ng/ml) as reported per included observational study [N = 24] with changes between pre-chemotherapy versus post-chemotherapy for a 6–60 month follow-up from the end of treatment, using a ball and stick regression model (burgundy line) with bin size 0–6 months and 12–60 months. (**B**) Cumulative study results with median AMH (ng/ml) per time-point values from 0 to 60 months. All data points subsequent to chemotherapy were normalized to reflect the same time trajectory from the end of treatment.

**Figure 3.**
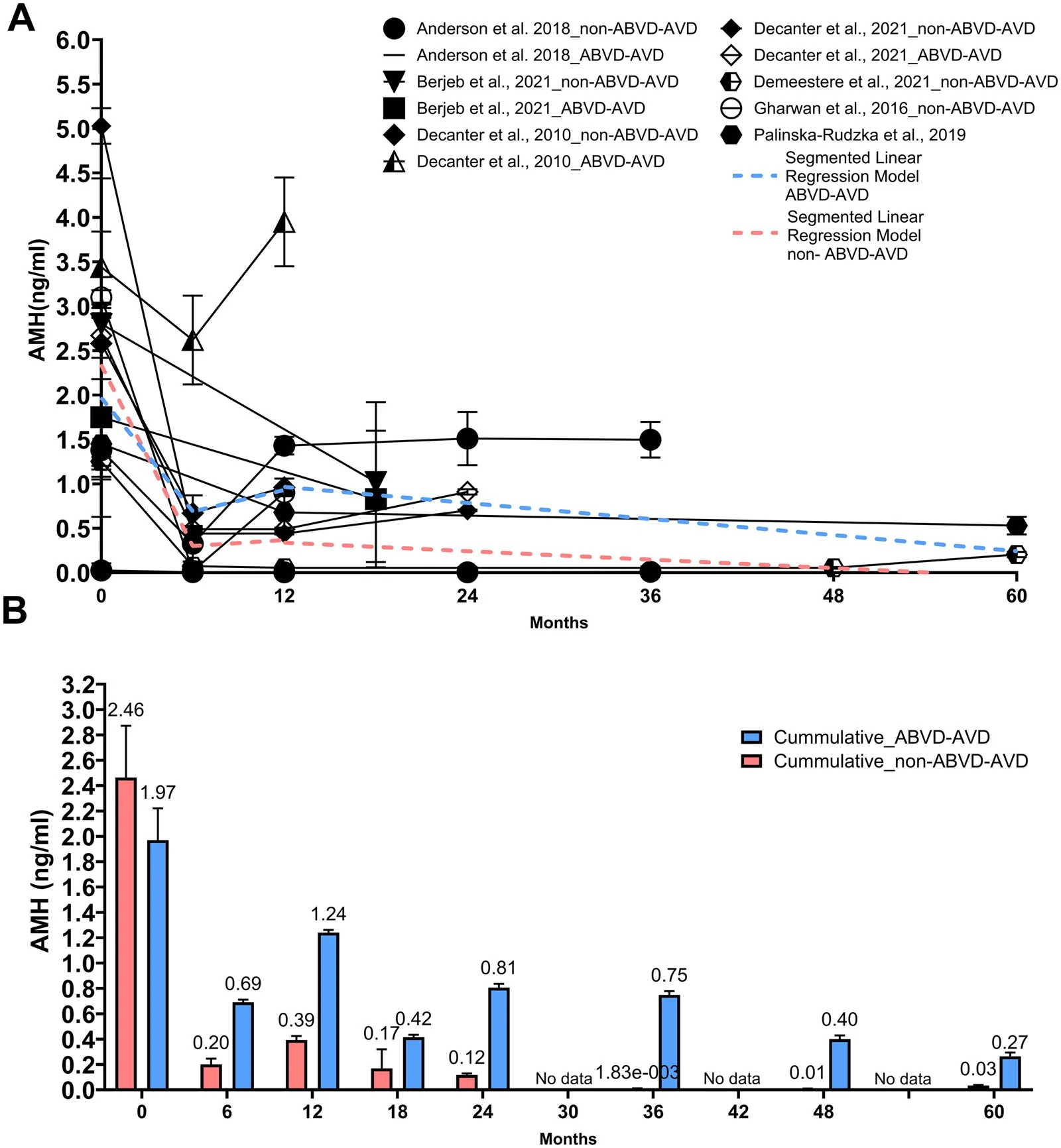
**AMH decline following chemotherapy in lymphoma patients.** (**A**) Data represent median and mean (SD) AMH values (ng/ml) as reported per included observational study [N = 7] with changes between pre-chemotherapy versus post-chemotherapy treatment for a 6–60 month follow-up. Patient populations per study, segregated by chemotherapy regimen-expected gonadotoxicity, and the ball and stick regression model of cumulative data per gonadotoxic treatment category (non-ABVD (pink line) versus ABVD-AVD (blue) with bin size 0–6 months and 12–60 months). (**B**) Cumulative study results with median AMH (ng/ml) per time-point values from 0 to 60 months. ABVD, treatment regimen including adriamycin, bleomycin, vinblastine, and dacarbazine; AMH, anti-Müllerian hormone.

**Figure 4.**
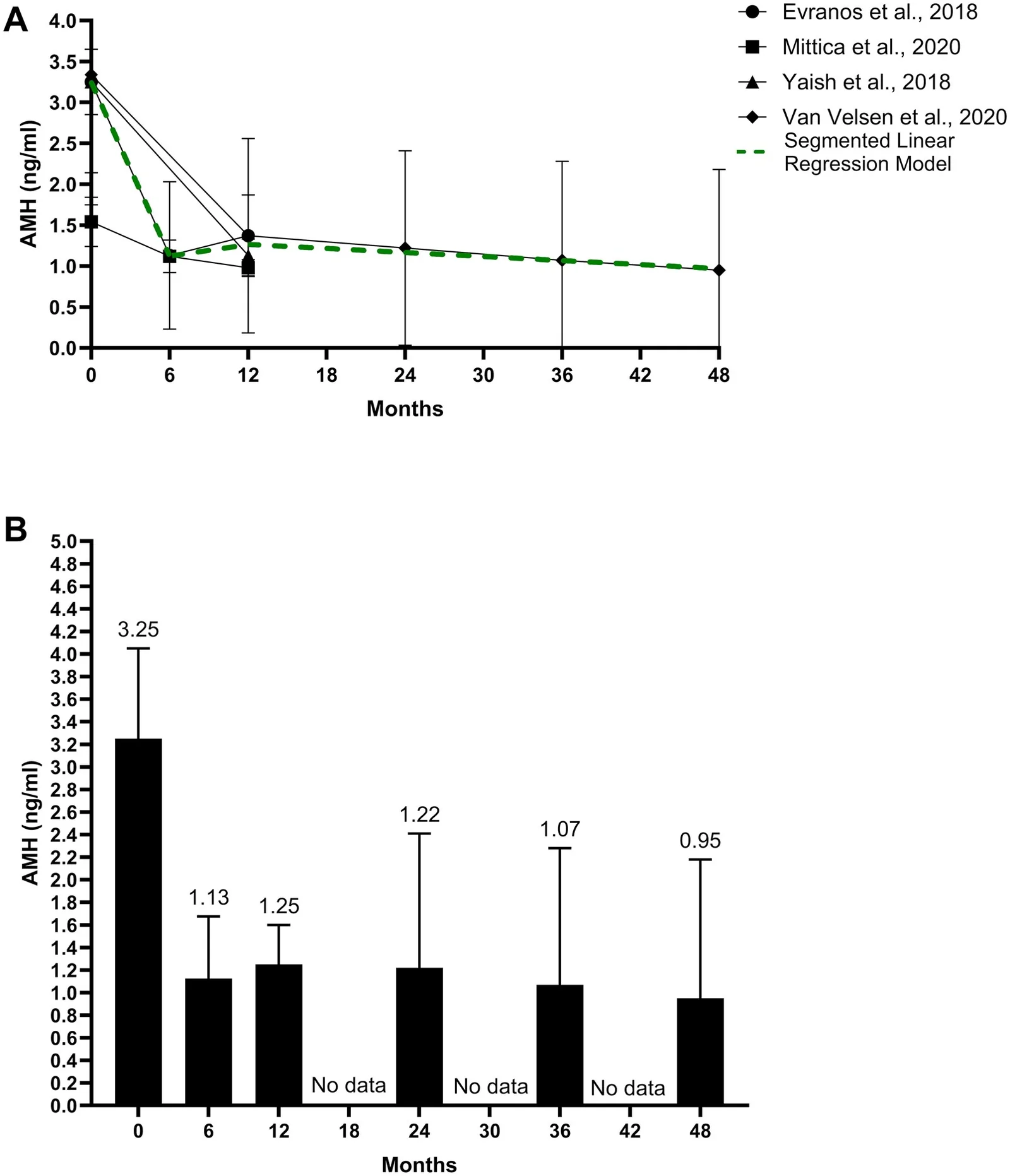
**AMH decline following treatment with RAI for thyroid cancer.** (**A**) Data represent median and mean (SD) AMH values (ng/ml) as reported per included observational study [N = 4] with changes between pre-chemotherapy versus post-chemotherapy treatment for a 6–48 month follow-up, using a ball and stick regression model of cumulative data (green line) with bin size 0–6 and 12–48 months. (**B**) Cumulative study results with median AMH (ng/ml) per time-point values from 0 to 48 months. AMH, anti-Müllerian hormone; RAI, radioactive iodine.

**Figure 5.**
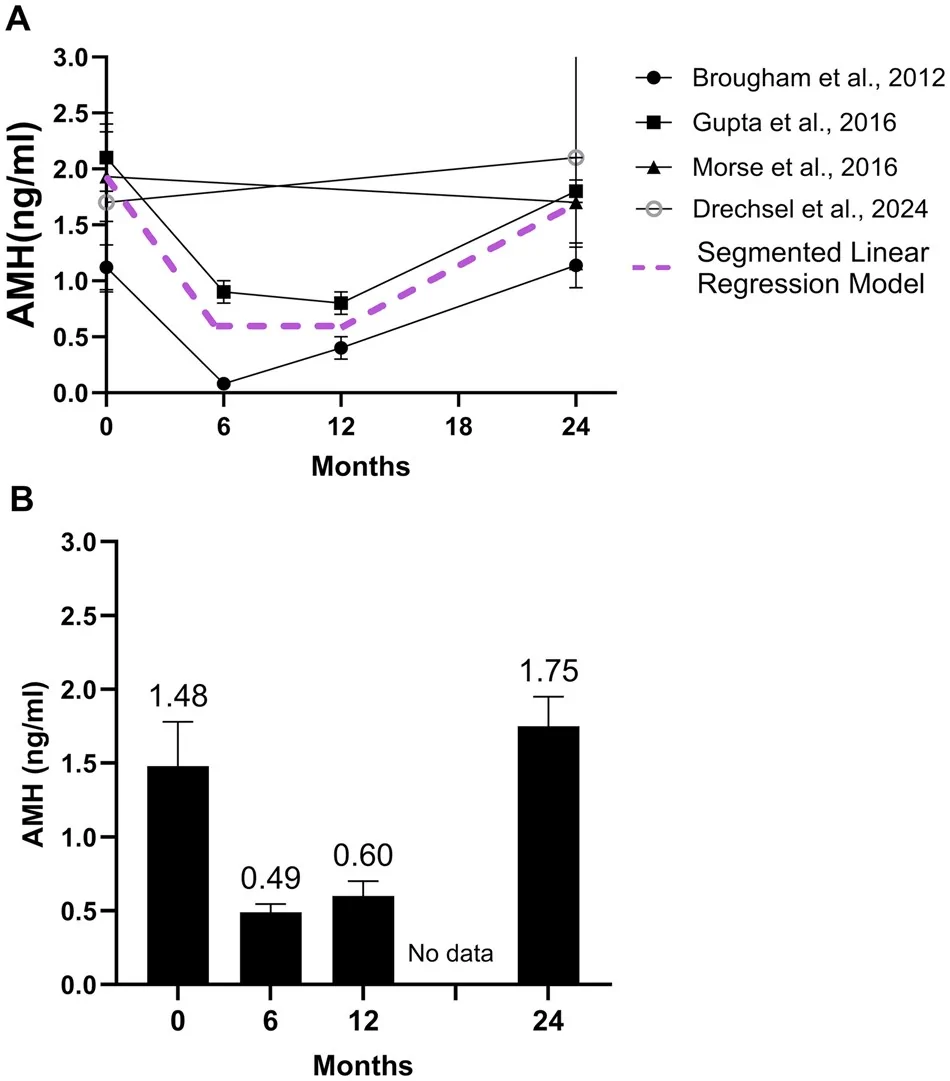
**AMH decline following chemotherapy for paediatric cancer (Ewings sarcoma, leukaemia, etc) patients.** (**A**) Data represent median and mean (SD) AMH values (ng/ml) as reported per included observational study [N = 4] with changes between pre-chemotherapy versus post-chemotherapy treatment for a 6–24 month follow-up; using a ball and stick regression model of cumulative data (purple line). (**B**) Cumulative results with median AMH (ng/ml) per time-point across studies. AMH, anti-Müllerian hormone.

**Table 1.** Summary of findings.

		Anticipated absolute effects* (95% CI)		No. of participants (No. of studies)	Certainty of the evidence (GRADE)	Comments
	Cohort age at time of diagnosis (median; IQR)	Baseline AMH (ng/ml)	AMH change after anti-cancer therapy change			
Breast cancer*	37.8 [34.0, 39.5]	2.0	Median change in AMH 76.4% lower (93.5 lower to 55.7)	2259 (24 studies)	⊕⊕⊕◯ Moderate^a^	a. Time points of AMH measurement, different AMH assays.
Lymphoma^*,ɫ^	26.0 [24.0, 26.9]	2.6	Median change in AMH 71.0% lower (95.5 lower to 3.6 higher)	431 (7 studies)	⊕⊕◯◯ Low^a, b, c^	a. Time points of AMH measurement, different AMH assays. b. Lymphomas grouped together. c. Different chemotherapy regimen, stratified in analysis
Lymphoma_ ABVD	24.0 [24.0, 26.0]	2.7	Median change in AMH 21.1% lower (81.6 lower to 14.8 higher)	146 (4 studies)	⊕⊕◯◯ Low^a^	a. Time points of AMH measurement, different AMH assays.
Lymphoma_ non-ABVD	26.3 [24.0, 28.0]	2.0	Median change in AMH 90.1 % lower (96.1 lower to 68.3 lower)	188 (6 studies)	⊕⊕⊕◯ Moderate^a^	a. Time points of AMH measurement, different AMH assays.
Thyroid cancers*	32.5 [26.0, 52.5]	3.3	Median change in AMH 57.9% lower (65.2 lower to 36.4 lower)	181 (4 studies)	⊕⊕⊕⊕ High^a^	a. Time points of AMH measurement, different AMH assays.
Adult population pre- and post-treatment AMH^^^	33.7 [26.8, 38.6]	2.2	Median change in AMH 71.0% lower (91.4 lower to 53.1 lower)	2871 (35 studies)	⊕⊕⊕◯ Moderate^a,c,d^	a. Time points of AMH measurement, different AMH assays c. Different chemotherapy regimens, stratified in analysis. d. Variability of cancers included.
Adult population longitudinal**	34.4 [23.0, 39.0]	N/A	Median drop/year in AMH since gonadotoxic exposure, 0.03–0.15 ng/ml till year 4.	2973 (20 studies)	⊕⊕⊕◯ Moderate^a,c,d^	a. Time points of AMH measurement, different AMH assays. c. Different chemotherapy regimens, stratified in analysis. d. Variability of cancers included.
Paediatric and AYA population pre- and post-treatment AMH^^^	9.0 [4.4, 14.3]	**1.9**	Median change in AMH 63.1% lower (64.3 lower to 61.9 lower)	176 (4 studies)	⊕◯◯◯ Very low^a,c,f^	a. Time points of AMH measurement, different AMH assays. c. Different chemotherapy regimens, stratified in analysis. f. small number of studies
Paediatric population longitudinal**	9.5 [7.0, 14.0]	N/A	Median drop/year in AMH since gonadotoxic exposure, 1.2–2.3 ng/ml till year 17.	1649 (21 studies)	⊕⊕⊕◯ Very low^a,c,d,e^	a. Time points of AMH measurement, different AMH assays. c. Different chemotherapy regimens, stratified in analysis. d. Variability of cancers included. e. Long duration since gonadotoxic exposure.

GRADE-Pro certainty in evidence summary. GRADE Working Group grades of evidence.

**High certainty:** we are very confident that the true effect lies close to that of the estimate of the effect.

**Moderate certainty:** we are moderately confident in the effect estimate: the true effect is likely to be close to the estimate of the effect, but there is a possibility that it is substantially different.

**Low certainty:** our confidence in the effect estimate is limited: the true effect may be substantially different from the estimate of the effect.

**Very low certainty:** we have very little confidence in the effect estimate: the true effect is likely to be substantially different from the estimate of effect.

* AMH change 12 months from the end of treatment.

** Nomogram population (Fig. 6).

^ Pre–post population (Figs 2, 3, 4, and 5).

All studies included were non-randomized.

ɫ Total number of participants with lymphoma exposed to chemotherapy, of whom 146 received ABVD, 188 received non-ABVD and 97 were uncategorizable in terms of chemotherapy regimen.

AMH, anti-Müllerian hormone; AYA, adolescents and young adults; ABVD, doxorubicin hydrochloride (Adriamycin), bleomycin sulfate, vinblastine sulfate, and dacarbazine.

### Risk of bias

Risk of bias was appraised across 79 studies (Supplementary Table S1; Supplementary Fig. S1). No study was rated as low overall risk of bias; 50.6% (n = 40) were rated high and 49.4% (n = 39) were rated moderate. The principal drivers of high risk were selection bias and confounding/co-intervention bias. These findings reflect the predominance of observational designs without pretreatment AMH baselines, particularly among studies contributing to the longitudinal data synthesis. Outcome measurement bias was rated low in 58.2% of studies (n = 46), largely due to the objectivity of AMH as a quantitative biomarker.

### AMH in patients with breast cancer

There were 24 studies that evaluated AMH levels before and after treatment for breast cancer (Table 2;  Fig. 2). The median age at the start of treatment was 37.8 [IQR: 34.0, 39.5]. Breast cancer patients exhibited a median AMH decline of 76.4% at 12 months (95% CI: −93.5 to −55.7; 24 studies; GRADE: moderate-certainty evidence), with some degree of recovery evident by 24 months. While it is likely that this is influenced by factors such as heterogeneity in the chemotherapy regimens and use of adjuvant endocrine therapies, results were generally presented in a way that made it impossible to include such factors in this analysis (Table 2;  Fig. 2).

**Table 2.** Characteristics of studies in breast cohorts included in the quantitative synthesis.

Publication	Study design	Adult/paediatric	Cohort size	Chemo regime	AMH assay	Timing of AMH assessment
Anderson and Cameron (2011)	Prospective longitudinal	Adult	42	Neo-adjuvant adriamycin and cyclophosphamide for six cycles (N = 3), CMF 6 cycles (N = 3) Anthracycline–CMF for 8–12 cycles (N = 25) Sequential anthracyclines and taxanes for eight cycles (N = 11)	ELISA (Beckman Coulter)	BL, FU 2, 3, 4, and 5 years (from diagnosis)
Anderson *et al.* (2017)*	Prospective longitudinal	Adult	101	Cyclophosphamide and/or anthracycline-containing regimens with or without a taxane for 6–8 cycles	Elecsys^®^ AMH (Roche Diagnostics)	BL, FU 12 and 24 months (from diagnosis)
Anderson *et al.* (2022)	Prospective cohort	Adult	206	Cyclophosphamide and anthracycline +- HER2 targeted therapy	Elecsys^®^ AMH (Roche Diagnostics)	BL, FU 6, 18, and 30 months (from diagnosis)
Ben-Aharon *et al.* (2015)	Prospective longitudinal	Adult	20	Trastuzumab-containing regimens and completed trastuzumab treatment for a total of 1 year (N = 7), regimens lacking cyclophosphamide (N = 4), Regimens lacking anthracyclines (N = 3)	Elecsys^®^ AMH (Roche Diagnostics)	BL, FU 0, 12, 24 months (from the end of treatment)
Berjeb *et al.* (2021)	Prospective longitudinal	Adult	23	FEC + TXT	Cobas E-411 Roche	BL, FU 16.8 ± 9.3 months (from the end of treatment)
Decanter *et al.* (2018)	Prospective longitudinal	Adult	32	Anthracyclines, i.e. three cycles of FEC100 (5-fluorouracil 500 mg/m^2^, epirubicin 100 mg/m^2^ and cyclophosphamide 500 mg/m^2^) and three cycles of taxanes (docetaxel 100 mg/m^2^).	picoAMH ELISA (Ansh)	BL, FU 3, 6, 9, and 12 months (from the end of treatment)
Dezellus *et al.* (2017)	Prospective cross-sectional	Adult	249	FEC (N = 12), FEC-T (N = 221), FEC-T (N = 1), AC-T (N = 1), Adri-blastin-T (N = 1), Taxanes (N = 14)	ELISA (Beckman Coulter—Immunotech)	BL, FU 6, 12, and 24 months (from the end of treatment)
Elgindy *et al.* (2013)	Prospective cross-sectional	Adult	100	5-flourouracil (500/m^2^), adriamycin (50 mg/m^2^), and cyclophosphamide (500 mg/m^2^) six cycles	Not reported	BL, FU 12 months (from the end of treatment)
Goldfarb *et al.* (2021)	Prospective cross-sectional	Adult	142	AC based (N = 106), CMF (N = 19) and tamoxfien alone (N = 17)	picoAMH ELISA (Ansh Labs)	BL, FU 12, 18, and 24 months (from the end of treatment)
Hamy *et al.* (2016)	Prospective cross-sectional	Adult	134	Anthracyclines and cyclophophamide-based regimen (N = 37, Taxanes containing regimen (N = 97)	ELISA (Beckman Coulter—Immunotech)	BL, FU 4–66 months (from the end of treatment)
Henry *et al.* (2014)	Prospective cross-sectional	Adult	27	ddAC (N = 2), ddAC + dd paclitaxel (N = 9), ddAC + weekly paclitaxel (N = 7), Docetaxel/cyclophosphamide (N = 9), Docetaxel/carboplatin (N = 1), Trastuzumab (N = 12)	Gen II ELISA (Beckman Coulter)	BL, FU 1 and 12 months (from the end of treatment)
Lambertini *et al.* (2019)	Prospective longitudinal	Adult	148	FEC (5 fluorouracil 500 mg/m^2^, epirubicin 100 mg/m^2^, cyclophosphamide 500 mg/m^2^) 3 or 6 cycles + 3 cycles of docetaxel (D, 100 mg/m^2^) (N = 127); Endocrine therapy: tamoxifen (±GnRH agonists) (N = 90)	Elecsys AMH (Roche Diagnostics)	BL, FU 1 and 3 years (from diagnosis)
Lambertini *et al.* (2025)	Prospective	Adult	194	Taxane and anthracycline/cyclophosphamide or carboplatin +- HER2 targeted therapy	Elecsys^®^ AMH (Roche Diagnostics)	BL, FU 6, 36 (from diagnosis)
Lee *et al.* (2020)	Prospective cross-sectional	Adult	67	Doxorubicin (60 mg/m^2^) and cyclophosphamide (600 mg/m^2^) for four cycles + four additional cycles of paclitaxel (175 mg/m^2^) or Cyclophosphamide (500 mg/m^2^), doxorubicin (50 mg/m^2^), and 5-fluorouracil (500 mg/m^2^) for six cycles	Gen II ELISA (Beckman Coulter)	BL, FU 1 year (from the end of treatment)
Li *et al*. (2020)	Prospective cross-sectional	Adult	144	TEC (docetaxel 75 mg/m^2^ + epirubicin 60 mg/m^2^ + CTX 500 mg/m^2^, D1) six cycles	Human AMH ELISA (Cusabio Biotech)	BL, FU 6 months (from the end of treatment)
Leonard *et al.* (2017)	Prospective cross-sectional	Adult	202	Cyclophosphamide and/or anthracycline-containing regimens with or without a taxane six to eight cycles	Elecsys AMH (Roche Diagnostics)	BL, FU 0, 9, 12, 24 months (from the end of treatment)
Lutchman Singh *et al.* (2007)	Prospective cross-sectional	Adult	35	E-CMF, FEC, Taxanes (docetaxel or paclitaxel), usually as part of a therapeutic trial (TANGO)	ELISA (Beckman Coulter—Immunotech)	BL, FU after resumption of menses
Palinska-Rudzka *et al.* (2019)	Prospective longitudinal	Adult	54	FECa, FEC + Docetaxel, ECb, EC + Doxetaxel, E-CMFc, additional paclitaxel or carboplatin	Gen II ELISA (Beckman Coulter)	BL, FU 1, 5 years (from diagnosis)
Silva *et al.* (2019)	Prospective longitudinal	Adult	38	Anthracycline-based (FEC/AC/EC) + sequential Paclitaxel/Docetaxel or Taxane based	Ultrasensitive ELISA (Ansh Labs)	BL, FU 1, 6 and >9 months up to 18 months (from the end of treatment)
Singh *et al.* (2025)	Prospective longitudinal	Adult	67	Alkylating (46/67) versus non-alkylating agents (21/67)	Roche Cobas e 801	BL, FU 6, 12 months (from the end of treatment)
Trapp *et al.* (2017)	Prospective cross-sectional	Adult	170	EC-Doc	not reported	BL, FU 24 months (from diagnosis)
Yu *et al.* (2010)	Prospective longitudinal	Adult	26	ACT, CMF, CAF	ELISA (Diagnostic Systems Laboratories)	BL, FU 6, 12, 24, 36, 52 weeks (from diagnosis)
Wenners *et al.* (2017)	Prospective longitudinal	Adult	51	Anthracycline-based chemotherapy, combinations with taxanes or anthracycline-free chemotherapy	Ultrasensitive ELISA (Ansh Labs)	BL, FU 6, 12, 24 months (from diagnosis)
Zhong *et al.* (2019)	Prospective cross-sectional	Adult	96	All: anthracycline-based therapy; 79% received therapy featuring either paclitaxel or docetaxel	Immunoassay (Beckman Coulter Unicel DX1800	BL, FU 6, 12 months (from the end of treatment)

AMH, anti-Müllerian hormone; BL, baseline; FU, follow-up; CMF, cyclophosphamide, methotrexate, and fluorouracil; HER2, Human Epidermal Growth Factor Receptor 2; FEC, Fluorouracil, Epirubicin, and Cyclophosphamide; FEC + TXT, Fluorouracil, Epirubicin, and Cyclophosphamide + Docetaxel; AC, Adriamycin (doxorubicin) and Cyclophosphamide; ddAC, Dose-dense Doxorubicin and Cyclophosphamide; AC-T, doxorubicin (Adriamycin) and cyclophosphamide paclitaxel (Taxol); CAF; cyclophosphamide, doxorubicin hydrochloride (Adriamycin), and fluorouracil; EC, Epirubicin and Cyclophosphamide; Ec-Doc, Epirubicin, Cyclophosphamide, and docetaxel.

### AMH in patients with lymphoma

Seven studies evaluated AMH levels before and after treatment for lymphoma (Table 3). The median age of this cohort was 26.0 [IQR: 24.0, 26.9]. Patients exposed to non-ABVD chemotherapy regimens experienced a median decline of 90.1% at 12 months (95% CI: −96.1 to −68.3; 6 studies; GRADE: moderate-certainty) (Fig. 3). ABVD chemotherapy also resulted in a decline in AMH but only by 21.1% (95% CI: −81.6 to +14.8; four studies; GRADE: low-certainty evidence) (Fig. 3).

**Table 3.** Characteristics of studies in the lymphoma cohorts included in the quantitative synthesis.

Publication	Study design	Adult/paediatric	Cohort size	Chemo regime	AMH assay	Timing of AMH assessment
Anderson *et al.* (2018)	Prospective longitudinal	Adult	67	Two cycles of ABVD and four cycles of AVD or two cycles of ABVD followed by BEACOPP-14 or esc BEACOPP four cycles	Elecsys AMH (Roche Diagnostics)	BL, FU 0, 1, 2, and 3 years
Berjeb *et al.* (2021)	Prospective longitudinal	Adult	27	BEACOPP alone, BEACOPP + ABVD or ABVD alone	Elecsys AMH (Roche Diagnostics)	BL, FU 6 and 18 months
Decanter *et al.* (2010)	Prospective longitudinal	Adult	30	ABVD, R-CHOP, ACVBP, BEAM, BEACOPP, and MINE (non ABVD)	21.7 ± 15.1 pmol/l	BL, FU 0, 3, 6, 9, and 12 months
Decanter *et al.* (2021)	Prospective longitudinal	Adolescent, young adult, Adult	122	ABVD, CHOP/R-CHOP, ACVBP/R-ACVBP, BEACOPP, COP/COPADEM, DHAP, BEAM, and MINE	Gen II ELISA (Beckman Coulter)	BL, FU 3, 6, 9, and 12 months
Demeestere *et al.* (2021)	Prospective longitudinal	Adult	145	Four cycles of Esc BEACOPP (n = 71), two cycles of esc BEACOPP, and ABVD (n = 75)	Elecsys AMH (Roche Diagnostics)	BL, FU 0, 1, 2, 3, 4, and 5 years
Gharwan *et al.* (2016)	Prospective longitudinal	Adult	28	DA-EPOCH-R	Gen II ELISA (Beckman Coulter)	BL, FU 4–18 months
Palinska-Rudzka *et al.* (2019)	Prospective longitudinal	Adult	12	ABVDd, ABVD + BEACOPPe, Chlorambucil, R-CHOPf	ELISA (Diagnostic Systems Laboratories)	BL, FU 1, 5 years

AMH, anti-Müllerian hormone; BL, baseline; FU, follow-up; ABVD, Adriamycin (doxorubicin), Bleomycin, Vinblastine, and Dacarbazine; AVD, doxorubicin, vinblastine, and dacarbazine; BEACOPP-14, bleomycin etoposide, doxorubicin, cyclophosphamide, vincristine, procarbazine and prednisolone; R-CHOP, rituximab, cyclophosphamide, doxorubicin, and vincristine, and prednisone; ACVBP, doxorubicin, cyclophosphamide, vindesine, bleomycin, prednisone; BEAM, carmustine, etoposide, cytarabine and melphalan; MINE, mesna, ifosfamide, novantrone (mitoxantrone) and etoposide; COP, cyclophosphamide, Oncovin and Prednisolone; COPADEM, Cyclophosphamide, Oncovin, Adriamycin, Methotrexate and Prednisolone; DHAP, dexamethasone, cytarabine, and Pcisplatin; DA-EPOCH-R, etoposide phosphate, prednisone, vincristine sulfate (Oncovin), cyclophosphamide, doxorubicin hydrochloride and rituximab.

### AMH in patients with thyroid cancer

Four studies evaluated AMH levels before and after treatment for thyroid cancer (Table 4). The median age of this cohort was 32.5 [IQR: 26.0, 52.5]. Thyroid cancer patients undergoing treatment with RAI exhibited an AMH decline of 57.9% at 12 months (95% CI: −65.2 to −36.4; four studies; GRADE: high-certainty evidence) (Fig. 4).

**Table 4.** Characteristics of studies in the thyroid cohorts included in the quantitative synthesis.

Publication	Study design	Adult/paediatric	Cohort size	RAI activity	AMH assay	Timing of AMH assessment
Evranos *et al.* (2018)	Prospective longitudinal	Adult	33	111.36 ± 27.3	ELISA (Cusabio)	BL, FU 3, 6, 9, and 12 months
Mittica *et al.* (2020)	Prospective cross-sectional	Adult	59	100.1 ± 117.4 (80; 60–100)	Immunoassay, manufacturer not reported	BL, FU 15.0–27.4 months
van Velsen *et al.* (2020)	Prospective cross-sectional	Adult	65	Single RAI group 50 (30–146), multiple RAI group 291 (284–293)	Gen II ELISA (Beckman Coulter)	BL, FU 6–9 months, then regular sampling up to 48 months
Yaish *et al.* (2018)	Prospective longitudinal	Adult	24	DTC 103 (53), Graves 13 (5)	Gen II ELISA (Beckman Coulter)	BL, FU 0, 3, 6, 9, and 12 months

AMH, anti-Müllerian hormone; BL, baseline; FU, follow-up; RAI, radioactive iodine; DTC, differentiated thyroid cancer.

### AMH in paediatric and adolescent and young adults cancer survivors

Four studies assessed paediatric and adolescent and young adults (AYA) cancer survivor cohorts, containing a variety of malignancies including Hodgkin lymphoma, sarcoma, leukaemia, Wilm’s tumour and germ cell tumours. The median age at time of diagnosis of this cohort was 9.0 (IQR: 4.4, 14.3). The AMH reduction at 12 months was 63.1% (95% CI: 64.3–61.9; four studies; GRADE: very low-certainty evidence), with good recovery seen at later time points; however, follow-up was restricted to 24 months for all included studies (Table 5;  Fig. 5).

**Table 5.** Paediatric, adolescent and young adult cohorts.

Publication	Study design	AYA/paediatric	Cohort size	Median age at diagnosis (years)	Cancer	AMH assay	Timing of AMH assessment
Brougham *et al.* (2012)	Prospective longitudinal	Paediatric	22	4.4	Hepatoblastoma, neuroblastoma, Langerhans cell histiocytosis, Germ cell tumour, Rhabdomyosarcoma, Wilms tumour, Hodgkin’s disease, T-cell lymphoma	Gen II ELISA (Beckman Coulter)	BL, FU 1- 12, >12 months
Gupta *et al.* (2016)	Prospective longitudinal	Paediatric	16	14.3	Leukaemia, lymphoma, and sarcoma	Gen II ELISA (Beckman Coulter)	BL, FU 6, 12 months
Mörse *et al.* (2016)	Prospective longitudinal	Paediatric	34	9.0	Wilms tumour, osteosarcoma, Ewing sarcoma	Gen II ELISA (Beckman Coulter)	BL, FU 3, 6, 9, 12, 15, 18, 21, 24, 27, 30, 32
Drechsel *et al.* (2024)	Prospective longitudinal	Paediatric	104	15.6	Classical Hodgkin lymphoma	Cobas, Roche Diagnostics	BL, FU 24

AMH, anti-Müllerian hormone; AYA, adolescent and young adult; BL, baseline; FU, follow-up.

### Longitudinal AMH assessment in adult and paediatric patients

There were 41 studies that evaluated longitudinal AMH levels at various time points following treatment in patients with different malignancies (Tables 6 and 7). These studies did not measure pre-treatment AMH levels.

**Table 6.** Adult studies included in the longitudinal graph.

Publication	Study design	Adult/paediatric	Cohort size	Cancer type	AMH assay	Time since diagnosis
Behringer *et al.* (2013)	Prospective cross-sectional	Adult	562	Lymphoma	Gen II ELISA (Beckman Coulter)	46 months
Demeestere *et al.* (2016)	Prospective longitudinal	Adult	67	Lymphoma	Elecsys AMH (Roche Diagnostics)	5–7 years
Di Paola *et al.* (2013)	Prospective longitudinal	Adult	63	HL, NHL and AML	Gen II ELISA (Beckman Coulter)	7/8 ± 5.2 years
Dillon *et al.* (2013)	Prospective longitudinal	Adult	84	Lymphoma, leukaemia, sarcoma, breast, Wilm’s and other	ELISA (Diagnostic Systems Laboratories)	14 years
Hamy *et al.* (2016)	Prospective cross-sectional	Adult	17	Breast	ELISA (Beckman Coulter—Immunotech)	59 months (range: 11–104)
Kim *et al.* (2018)	Prospective cross-sectional	Adult	82	Breast	ELISA (USCN Life Science)	61.8 months (range 9.1–71.5
Lambertini *et al.* (2019)	Prospective longitudinal	Adult	148	Breast	Elecsys AMH (Roche Diagnostics)	3 years
Lee *et al.* (2018)	Prospective cross-sectional	Adult	69	Breast	Gen II ELISA (Beckman Coulter)	mean: 737 days, range: 486–1074 days
Leonard *et al.* (2017)	Prospective cross-sectional	Adult	221	Breast	Elecsys AMH (Roche Diagnostics)	24 months
Loubersac *et al.* (2020)	Prospective Cross-sectional	Adult	238	Breast	Immunotech Beckman Coulter ELISA	2 years
Malisic *et al.* (2018)	Prospective cross-sectional	Adult	40	Breast	ELISA (AnshLabs)	31 months (range: 1–66 months).
Morarji *et al.* (2017)	Prospective longitudinal	Adult	100	Breast	Gen II ELISA (Beckman Coulter)	2 years after chemotherapy (range: 0.04–13.5 years
Nitzschke *et al.* (2010)	Prospective longitudinal	Adult	20	Lymphoma	ELISA (Diagnostic Systems Laboratories)	30 months
Palinska-Rudzka *et al.* (2019)	Prospective cross-sectional	Adult	20	Breast	ELISA (Diagnostic Systems Laboratories)	5 years
Parissone *et al.* (2021)	Prospective longitudinal	Adult	55	Mixed	Gen II ELISA (Beckman Coulter)	Median 10.2 (0.8–24.2), mean 10.2 years
Roshandel *et al.* (2021)	Prospective cross-sectional	Adult	138	ALL	Not reported	Median years (IQR) 21.9 (10.4)
Shandley *et al.* (2017)	Prospective longitudinal	Adult	108	Breast	Ultrasensitive ELISA and pico AMH ELISA (Ansh Labs)	Seven years (interquartile range [IQR] 5–10).
Su *et al.* (2010)	Prospective longitudinal	Adult	127	Breast	ELISA (Diagnostic Systems Laboratories)	5.2 years since chemotherapy (range: 1.0–7.6)
Su *et al.* (2020)	Prospective longitudinal	Adult	763	Leukaemia, sarcoma, breast, GI, lymphoma, thyroid	ELISA for BDS (Ansh Labs)	7.4 (4.8) years
Wenners *et al.* (2017)	Prospective longitudinal	Adult	51	Breast	Ultrasensitive ELISA (Ansh Labs)	24 months

AMH, anti-Müllerian hormone; HL, hodgkin lymphoma; NHL, non-Hodgkins lymphoma; AML, acute myeloid leukaemia.

**Table 7.** Paediatric studies included in the longitudinal graph.

Publication	Study design	Adult/paediatric	Cohort size	Cancer type	AMH assay	Time since diagnosis* (Years)
Cameron *et al.* (2019)	Prospective longitudinal	Paediatric	170	Cancer treatments: 44 (85%) received treatment with alkylators, seven (4%) received pelvic radiation, and 35 (20%) had a history of bone marrow transplant [BMT; 15 with total body irradiation (TBI)	picoAMH ELISA (Ansh Labs)	9.9 [5.1, 16.0]
Charpentier *et al.* (2014)	Prospective longitudinal	Paediatric	66	Leukemia, Hodgkin lymphoma (HL), or sarcoma	ELISA (Beckman Coulter—Immunotech)	11.5 [1.4, 25.1]
Elchuri *et al.* (2016)	Prospective longitudinal	Paediatric	49	Leukemia, lymphoma and renal tumours	Gen II ELISA (Beckman Coulter)	7.5
Elitzur *et al.* (2021)	Prospective longitudinal	Paediatric	42	ALL	Gen II ELISA (Beckman Coulter)	20.6 [5.3, 33.2]
Hamre *et al.* (2012)	Prospective cross-sectional	Paediatric	62	Lymphoma	ELISA (Beckman Coulter)	18.1 [7.1, 36.2]
Jin *et al.* (2023)	Prospective cross-sectional	Paediatric	69	Leukaemia, lymphoma, CNS tumour, embryonal tumour, bone and soft tissue sarcoma, ovarian tumour	Elecsys AMH (Roche Diagnostics)	10.8 [1-16.5]
Jingrukwong *et al.* (2025)	Prospective cross-sectional	Paediatric	36	ALL, AML, Burkitt, ALLHR	Elecsys AMH (Roche Diagnostics) Gen II ELISA (Beckman Coulter)	4.4 [2.0, 6.5]
Krawczuk-Rybak *et al.* (2013a)	Prospective longitudinal	Paediatric	83	Hodgkin lymphoma, Wilms tumour, soft tissue sarcoma, STS, neuroblastoma, germinal tumours, acute myeloblastic leukemia, acute lymphoblastic leukaemia, chronic myeloblastic leukemia, and non-Hodgkin lymphoma	ELISA (Beckman Coulter—Immunotech)	6.03 [3.82, 9.11]
Krawczuk-Rybak *et al.* (2013b)	Prospective longitudinal	Paediatric	33	Hodgkin lymphoma, soft tissue sarcoma, Wilms tumour, neuroblastoma (stage II), germ tumours	ELISA (Beckman Coulter—Immunotech)	6.05 [3.0, 9.34] *(high-risk group)*, 6.64 [2.0, 3.89] *(medium/low-risk group)*
Krawczuk-Rybak *et al.* (2019)	Prospective longitudinal	Paediatric	25	ALL	ELISA (Beckman Coulter—Immunotech)	8.63 *(SD 4.73)*
Lie Fong *et al.* (2009)	Prospective cross-sectional	Paediatric	185	Leukaemia group (ALL and NHL), AML, HL, Neuroblastoma, sarcoma, Wilms tumour, LCH and other	ELISA (Beckman Coulter—Immunotech)	20
Lunsford *et al.* (2014)	Prospective longitudinal	Paediatric	53	Leukemia, lymphoma, Wilm’s tumour, Brain tumour, Sarcoma, Neuroblastoma and other	Gen II ELISA (Beckman Coulter)	7.5 [3.3–10.4]
Miyoshi *et al.* (2013)	Prospective longitudinal	Paediatric	53	solid tumour, haematological and brain tumour	ELISA (Beckman Coulter)	8.8 [2.3–26.1
Nielsen *et al.* (2013)	Prospective longitudinal	Paediatric	71	Leukaemia, Wilm’s tumour, Neuroblastoma, Ewing’s sarcoma, sarcoma, malignant teratoma,	ELISA (Beckman Coulter—Immunotech)	3.5 [3–20.0]
Nies *et al.* (2020)	Prospective cross-sectional	Paediatric	56	Differentiated thyroid cancer	Elecsys AMH (Roche Diagnostics)	15.4 [IQR 8.3–24.7]
Nilsson *et al.* (2022)	Prospective longitudinal	Paediatric	54	Multiple, SCT	Not reported	11 [1-23]
Nyström *et al.* (2019)	Prospective longitudinal	Paediatric	120	Leukaemia, brain tumour, lymphoma, sarcoma, Wilm’s (Krawczuk-Rybak, Płonowski *et al.* 2019)tumour, Ovarian tumour and other	ELISA (manufacturer not stated)	25.4 [11.6–41.3]
Signorino *et al.* (2025)	Retrospective cohort	Paediatric	95	Leukaemia, lymphoma, bone and soft tissue sarcoma, myelodysplastic syndrome	Cobas 8000 (Roche Diagnostics)	4.4 [0.1, 11.4]
Thomas-Teinturier *et al.* (2015)	Prospective longitudinal	Paediatric	105	ALL, HL, NHL, Sarcoma, soft tissue, neuroblastoma, other	Gen II ELISA (Beckman Coulter—Immunotech)	16
van Beek *et al.* (2007)	Prospective longitudinal	Paediatric	30	Lymphoma	ELISA (Diagnostic Systems Laboratories)	11.4 [5.7-17.7]
van der Kooi *et al.* (2017)	Prospective longitudinal	Paediatric	192	Leukaemia, lymphoma sarcoma, renal tumour, neuroblastoma, germ cell tumour, brain tumour and other	Ultrasensitive ELISA (Immunotech Coulter)	15.8 [5.0–43.2]

AMH, anti-Müllerian hormone; BL, baseline; FU, follow-up; HL, Hodgkins lymphoma; NHL, non-Hodgkins lymphoma; ALL, acute lymphoblastic leukaemia; AML, acute myeloid leukaemia; ALL-HR, high-risk acute lymphoblastic leukemia; SCT, stem cell transplant; LHS, Langerhans Cell Histiocytosis.

* All values depicted are in median and range format unless otherwise stated.

The adult cancer population (20 studies, N = 2973) had a median age of 34.4 [IQR: 23.0, 39.0] and were median of 6.0 [IQR: 2.5, 7.2] years from the end of treatment. A nomogram of AMH values from women attending UK fertility clinics was used as the adult non-cancer comparator group (Nelson *et al.*, 2014). Compared to the non-cancer group, adult cancer survivors showed a trend towards higher AMH levels in the first 6 years after cancer treatment; however, this difference was not statistically significant, and both groups followed a similar trajectory of gradual decline thereafter (Fig. 6A).

**Figure 6.**
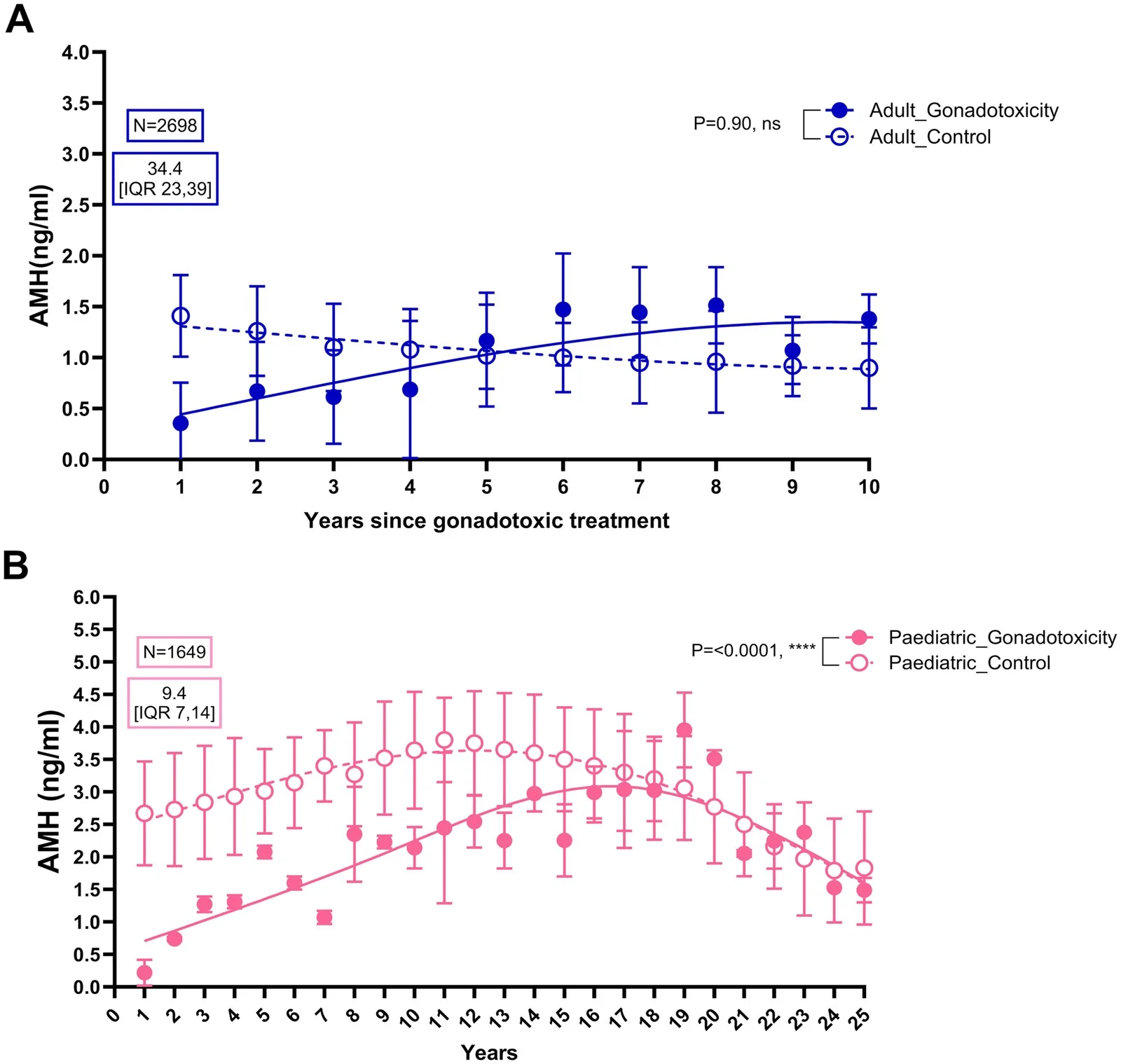
**Longitudinal AMH levels in adult (A) and paediatric (B) populations post-chemotherapy.** The trajectory of AMH decline varies with age at treatment initiation (adult: blue line; paediatric: pink line, with 95% error bars above and below each data point). Single AMH (ng/ml) measurement years are given for 1 to 25 years post-treatment. Spline Slope comparison between populations (control versus gonadotoxicity) and paired *t*-tests were used. Control populations were derived from Nelson *et al*. (2014) and Hagen *et al*. (2023). AMH, anti-Müllerian hormone; IQR, interquartile range.

The paediatric cancer longitudinal cohort (21 studies, N = 1649) had a median age of 9.5 [IQR: 7.0, 14.0] and a median of 10.8 [IQR: 7.5, 15.8] years from the end of treatment. The paediatric non-cancer comparator group was obtained from an analysis that reported AMH data from infancy to adolescence, with a median age of 15.9 years (Hagen *et al.*, 2023). The paediatric patients exhibited a relatively greater potential for AMH recovery at later time points, whereas adult patients demonstrated a more sustained decline (Fig. 6B).

Overall, long-term AMH trajectories in both adult and paediatric groups appear relatively stable and closely aligned with age-matched non-cancer controls. However, interpretation is limited by heterogeneity in the exclusion of individuals with POI and by variable definitions of ovarian function across studies. Both adult and paediatric survivors who maintain detectable ovarian activity do not appear to experience an accelerated decline in AMH over time (Fig. 6A and B). Furthermore, paediatric cancer survivors exhibited a similar increase in AMH to that observed in healthy controls from childhood to the adolescent years.

## Discussion

### Main findings

This study demonstrates the impact of chemotherapy on ovarian reserve, as measured by AMH levels, across a range of malignancies and in both girls and adult women. A key finding is that, in all four cohorts (breast, lymphoma, thyroid, and paediatric/AYA mixed cancers), AMH levels were increased at 12–24 months from the end of treatment compared with levels taken at 6 months. This supports previous observations that early post-treatment AMH assessment may underestimate long-term ovarian function (Anderson and Cameron, 2011; Cameron *et al.* 2019; Su *et al.* 2020; Decanter *et al.* 2021; Anderson *et al.*, 2022; Yano Maher *et al.*, 2025). The observed temporal pattern in the AMH recovery at 12 and 24 months post-treatment likely reflects the underlying dynamics of follicular development. AMH is produced by granulosa cells of pre-antral and small antral follicles and therefore reflects the pool of actively growing follicles rather than the dormant primordial reserve (Carlsson *et al.*, 2006) and predominantly those antral follicles of 5–8 mm diameter (Weenen *et al.*, 2004; Jeppesen *et al.*, 2013). In the immediate post-treatment period, AMH levels are typically low due to the preferential destruction of growing follicles by cytotoxic therapies, which target rapidly proliferating cells (Meirow *et al.*, 2010). This explains the marked suppression of AMH and the frequent occurrence of treatment-related amenorrhoea during and shortly after chemotherapy (Anderson and Cameron, 2011; Jacobson *et al.*, 2016; Dezellus *et al.*, 2017). If follicular recruitment from the primordial pool is preserved, there is an inherent delay before this is reflected in circulating AMH levels. It has been estimated that the transition from primordial follicle activation to the pre-antral stage may take over 300 days, which may explain the delayed recovery in AMH observed in the months following completion of treatment (Dezellus *et al.*, 2017; Palinska-Rudzka *et al.*, 2019). Thus, early changes in AMH following chemotherapy primarily reflect acute suppression of follicular activity rather than permanent depletion of the ovarian reserve. As such, AMH measurements obtained within the first 6 months post-treatment should be interpreted with caution, and longitudinal assessment provides a more accurate representation of residual ovarian function.

### Treatment-specific effects

The magnitude of AMH decline varied across malignancies and treatment regimens. Patients treated for breast cancer demonstrated an almost 75% reduction in AMH, consistent with prior studies, which have categorized breast cancer chemotherapy regimens as intermediate to high risk for permanent amenorrhoea in women aged 30 and over (Reynolds and McKenzie, 2023). However, interpretation is limited by the potential confounding effects of endocrine therapy, including tamoxifen, aromatase inhibitors, and GnRH analogues, which may independently influence ovarian function and menses and potentially AMH levels (Lambertini *et al.*, 2019). As treatment-specific data were not consistently available, the magnitude of chemotherapy-related AMH decline may be overestimated in this group.

Among lymphoma patients, those treated with non-ABVD regimens experienced the most pronounced decline, with a median reduction of 90% at 12 months, whereas those treated with ABVD exhibited the least decline (21%). The non-ABVD group likely represents a heterogeneous mix of treatment regimens with varying degrees of gonadotoxicity. Many of these protocols include alkylating agents, which are known to be highly gonadotoxic, such as cyclophosphamide, ifosfamide, and melphalan (Decanter *et al.*, 2018; Decanter *et al.*, 2021). Examples include CHOP/R-CHOP, ACVBP/R-ACVBP, BEACOPP, COP/COPADEM, DHAP, BEAM, and MINE (Decanter *et al.*, 2021), where the cumulative exposure to alkylating agents within these regimens is likely to contribute to the marked reduction in AMH observed in this group. Newer treatment regimens, such as BrECADD, which was evaluated in the recent HD21 clinical trial, may have a relatively lower impact on ovarian reserve, although a substantial decline in AMH was still observed (Ferdinandus *et al.*, 2025). Given the timeframe of the included studies, it is unlikely that BrECADD had been used in the studies included in our analysis, and the observed effects in the non-ABVD group may predominantly reflect older, more gonadotoxic regimens such as escalated BEACOPP in Hodgkin lymphoma and R-CHOP in non-Hodgkin lymphoma.

The thyroid cancer cohort treated with RAI showed a nearly 60% reduction in AMH, adding further evidence for the potential gonadotoxicity of RAI treatment. Notably, this cohort showed relatively low heterogeneity compared with other cancer groups and was graded as high certainty of evidence, strengthening confidence in this finding. The consistency observed across studies may reflect the relatively standardized nature of RAI dosing and treatment protocols (Pacini *et al.*, 2022), in contrast to the greater variability seen with multi-agent chemotherapy regimens. Although the gonadotoxic effects of RAI are generally considered less pronounced than those of alkylating chemotherapy, the observed decline in AMH suggests that RAI may still exert a clinically meaningful impact on ovarian function. Clinicians should be aware of the potential implications of RAI on ovarian reserve when counselling patients, particularly those with a low pre-treatment AMH and those in their later reproductive years.

The impact of newer systemic therapies, particularly immune checkpoint inhibitors, remains incompletely understood. There are data in animal models indicating direct ovarian toxicity (Winship *et al.*, 2022), and these agents may also affect reproductive function indirectly through endocrine disruption, including immune-related hypophysitis (Duma and Lambertini, 2020). However, the proportion of patients receiving concurrent immunotherapy was not reported in all the included studies, and their contribution to observed AMH trajectories remains uncertain.

### Long-term AMH trajectories and reproductive implications

We also examined long-term AMH trajectories in cancer survivors who retained ovarian function after treatment. In both childhood/adolescent and adult survivors, AMH levels remained relatively stable over time and closely mirrored those of age-matched healthy controls. These findings are consistent with previous studies demonstrating that, although AMH levels are reduced following treatment, the rate of decline thereafter may parallel normal reproductive ageing (van der Kooi *et al.* 2017; Cameron *et al.*, 2019; Su *et al.*, 2020). Thus, survivors who do not experience early ovarian insufficiency are unlikely to undergo accelerated depletion of their ovarian reserve and may follow a typical trajectory of reproductive ageing.

A non-significant trend towards higher AMH levels was observed in adult survivors within the first 6 years following treatment. This likely reflects recovery of the growing follicle pool following the acute suppressive effects of chemotherapy with the re-establishment of the normal hierarchy of different growing follicle populations rather than true restoration of ovarian reserve. The exclusion or under-representation of individuals with POI may also result in overestimation of AMH levels and more favourable long-term trajectories in this subgroup.

AMH levels in prepubertal girls are physiologically lower than in adult women and vary with age and pubertal status. Although we used published paediatric reference cohorts as comparators (Hagen *et al.*, 2023), the reliability of these control groups may be limited by differences in assay platforms, population characteristics, and study design. In addition, longitudinal data in healthy paediatric populations remain relatively sparse, particularly across the transition from childhood to adolescence. These factors introduce uncertainty when comparing AMH trajectories between paediatric cancer survivors and controls.

### Strengths and limitations

This is the first comprehensive quantitative analysis of AMH trajectory after cancer treatment encompassing a wide range of malignancies and chemotherapy regimens, improving the generalizability of our findings. The estimation of median change in AMH from pretreatment to 12 months post-treatment across cancer cohorts provides clinically relevant reference data that may help contextualize post-treatment ovarian reserve and the likelihood of residual ovarian function following cancer therapy. The application of digital tools such as GraphReader and PlotDigitizer enabled the integration of previously non-quantifiable datasets, enhancing the completeness of our meta-synthesis. However, these methods may introduce measurement error, as the accuracy of extracted values depends on the quality and resolution of the original figures. To mitigate this, each data point was extracted three times, and the mean of these measurements was used for analysis.

The role of the chemotherapy regimen in determining long-term ovarian function is critical, yet heterogeneity in treatment protocols and limited reporting across malignancy types made cross-cohort comparisons challenging. While we were able to compare ABVD and non-ABVD regimens within the lymphoma cohorts (while recognizing heterogeneity in the non-ABVD group), we could not evaluate regimen-specific effects in the other cancer types. Additionally, variability in AMH assay platforms, inconsistent reporting on the use of ovarian suppression agents such as GnRHa, and differences in follow-up duration are likely to have contributed to the heterogeneity observed in AMH trajectories.

A further limitation is that many studies excluded individuals who developed POI and had no detectable ovarian function post-treatment. While this was necessary to examine long-term AMH recovery patterns, it may over-represent favourable outcomes and limit the generalizability of our findings to all cancer survivors.

Finally, the relatively short follow-up duration in several studies may underestimate the potential for delayed ovarian recovery, particularly in younger patients. Post-treatment AMH trajectories in cancer survivors are heterogeneous and may include patterns of initial profound suppression followed by gradual recovery over 2–3 years rather than months (Su *et al.*, 2020). In younger women, the residual follicular pool may remain dormant or functionally suppressed in the early post-treatment period, with recovery of measurable AMH occurring well beyond the first year after therapy. Studies with limited follow-up may therefore misclassify transient ovarian suppression as permanent ovarian insufficiency. Furthermore, the use of hormone replacement therapy may mask the return of endogenous ovarian function, thereby delaying recognition of recovery and complicating the interpretation of ovarian reserve markers.

### Implications for research and clinical practice

Clinicians should exercise caution when interpreting AMH results obtained within the first 6–12 months after gonadotoxic treatment and avoid relying on isolated early measurements to make definitive conclusions regarding ovarian reserve or to diagnose ovarian insufficiency. Table 1, which summarizes the median change in AMH from pretreatment to 12 months post-treatment across cancer cohorts, may be used as a reference to support clinical decision-making. These estimates can help clinicians identify patients who are more likely to retain ovarian function after treatment and provide informed counselling regarding expected post-treatment ovarian reserve.

From a research perspective, longer-term longitudinal studies extending beyond 5–10 years after treatment and after more precisely defined treatment exposures are needed to determine whether AMH trajectories can reliably predict clinically meaningful outcomes such as age at menopause and overall reproductive lifespan. Importantly, future studies should include individuals who develop POI as well as those with preserved ovarian function to minimize selection bias and better characterize the full spectrum of reproductive outcomes following cancer treatment.

Greater consistency in the reporting of chemotherapy regimens, cumulative gonadotoxic exposure, and the use of concurrent ovarian suppression or other hormonal and immune therapies are also essential to improve comparability across studies.

Finally, the development of personalized predictive tools incorporating pretreatment AMH, age at treatment, and treatment-related gonadotoxicity could substantially improve fertility preservation decision-making by identifying individuals at highest risk of ovarian insufficiency who are most likely to benefit from intervention, while reducing unnecessary fertility preservation in those with a low likelihood of future need. This is particularly relevant in the context of an expanding population of cancer survivors and individuals exposed to gonadotoxic treatments for benign conditions, where judicious use of fertility preservation resources is increasingly important.

## Conclusion

This study quantifies the decline in AMH following chemotherapy across a range of cancer types and regimens and provides estimates of long-term AMH trajectories in female cancer survivors. Among those who retained ovarian function post-treatment, AMH levels typically recovered within 12–24 months from the end of treatment and remained stable thereafter in both adult and paediatric populations, closely resembling those of age-matched healthy controls.

These findings suggest that early post-treatment AMH measurements may underestimate residual ovarian function and that longitudinal assessment is more informative for clinical counselling. However, AMH reflects the growing follicle pool and does not directly predict fertility or time to pregnancy. Even in the presence of apparent recovery, the overall reproductive lifespan may remain reduced due to partial depletion of the primordial follicle pool. The addition of clinical and biochemical markers, including menstrual cycle patterns and FSH levels, would provide a more comprehensive assessment of ovarian function in cancer survivors.

Overall, these findings offer valuable insights into the residual reproductive lifespan following cancer treatment and underscore the value of AMH as a biomarker for long-term ovarian function. However, given the variability in individual recovery patterns and the absence of robust predictive models, ongoing monitoring of ovarian function and a cautious individualized approach remain essential when considering fertility preservation options and reproductive decision-making.

## Supplementary Material

dmag019_Supplementary_Data

## Data Availability

The data underlying this article are available in the article and in its online supplementary material.
