# Supplementary material for: Assessing the reproductive lifespan of female cancer survivors using AMH: a meta-synthesis of longitudinal studies
Source: Hum Reprod Update. 2026 Jul 24;32(6):735–56. doi: 10.1093/humupd/dmag019 (PMC13731750; doi:10.1093/humupd/dmag019)
Supplement: Supplementary file 1 [file dmag019_supplementary_data.docx]

Title: Assessing the reproductive lifespan of female cancer survivors using AMH: a meta-synthesis of longitudinal studies

**Running title:** AMH trajectories in female cancer survivors

**Full author list:** Neerujah Balachandren†^1^*, Stavroula L Kastora†^1^, Richard A Anderson^2^, Clara Luehwink^1^, Melanie C Davies^1^, Stuart A Lavery^1^, Dimitrios Mavrelos^1^ and Ephia Yasmin^1^

## Affiliations:

## 1. University College London Hospital, London, UK

## 2.  Centre for Reproductive Health, Institute for Regeneration and Repair, University of Edinburgh, Edinburgh, UK.

## Correspondence address: Reproductive Medicine Unit, Elizabeth Garrett Anderson Wing, University College Hospital, 25 Grafton Way, London, WC1E 6DB. UK. Email: [n.balachandren@nhs.net](mailto:n.balachandren@nhs.net) <https://orcid.org/0000-0003-3086-5235>

†Neerujah Balachandren and Stavroula L. Kastora contributed equally to this work.

**Table of contents:**

Supplementary Data File S1. Search terms.

Supplementary Table S1. Risk of bias appraisal.

Supplementary Figure S1: Risk of bias summary figure.

Supplementary Table S2. Crude data per graph.

Supplementary Table S3. Mixed population studies or those that did not offer extractable data for inclusion in the longitudinal cohorts but were included in the systematic review.

Supplementary Data File S2. Supplementary references

**Supplementary Data File S1. Search terms**

1. Ovid MEDLINE(R)

Database: Ovid MEDLINE(R) ALL

1. exp Primary Ovarian Insufficiency/

2. exp Menopause, Premature/

3. ((premature or primary) adj3 ovarian adj3 (insufficien* or failure*)).ti,ab.

4. (premature menopaus* or gonadotoxic*).ti,ab.

5. 1 or 2 or 3 or 4

6. exp Anti-Mullerian Hormone/

7. (AMH or antimullerian* or "anti-mullerian hormone*" or "anti mullerian hormone*" or "anti-muellerian hormone*" or "mullerian inhibiting substance" or "mullerian inhibiting hormone" or "anti-mullerian factor").ti,ab.

8. 6 or 7

9. exp Neoplasms/

10. (cancer* or neoplas* or malignan* or tumor* or tumour* or carcinoma* or lymphoma* or leukemia* or leukaemia* or sarcoma* or blastoma* or melanoma*).ti,ab.

11. 9 or 10

12. 5 and 8 and 11

2. Embase via Ovid

2. Database: Embase

Platform: Ovid

1. exp premature menopause/

2. ((premature or primary) adj3 ovarian adj3 (insufficien* or failure*)).ti,ab.

3. (premature menopaus* or gonadotoxic*).ti,ab.

4. 1 or 2 or 3

5. exp anti mullerian hormone/

6. (AMH or antimullerian* or "anti-mullerian hormone*" or "anti mullerian hormone*" or "anti-muellerian hormone*" or "mullerian inhibiting substance" or "mullerian inhibiting hormone" or "anti-mullerian factor").ti,ab.

7. 5 or 6

8. exp neoplasm/

9. (cancer* or neoplas* or malignan* or tumor* or tumour* or carcinoma* or lymphoma* or leukemia* or leukaemia* or sarcoma* or blastoma* or melanoma*).ti,ab.

10. 8 or 9

11. 4 and 7 and 10

3. Cochrane Central Register of Controlled Trials (CENTRAL)

Database: Cochrane Central Register of Controlled Trials (CENTRAL)

#1 MeSH descriptor: [Primary Ovarian Insufficiency] explode all trees

#2 MeSH descriptor: [Menopause, Premature] explode all trees

#3 ((premature or primary) near/3 ovarian near/3 (insufficien* or failure*)):ti,ab,kw

#4 (premature menopaus* or gonadotoxic*):ti,ab,kw

#5 #1 OR #2 OR #3 OR #4

#6 MeSH descriptor: [Anti-Mullerian Hormone] explode all trees

#7 (AMH or antimullerian* or "anti-mullerian hormone" or "anti mullerian hormone" or "anti-muellerian hormone" or "mullerian inhibiting substance" or "mullerian inhibiting hormone" or "anti-mullerian factor"):ti,ab,kw

#8 #6 OR #7

#9 MeSH descriptor: [Neoplasms] explode all trees

#10 (cancer* or neoplas* or malignan* or tumor* or tumour* or carcinoma* or lymphoma* or leukemia* or leukaemia* or sarcoma* or blastoma* or melanoma*):ti,ab,kw

#11 #9 OR #10

#12 #5 AND #8 AND #11

4, Google Scholar

Database: Google Scholar

Search 1:

"anti-Mullerian hormone" cancer/neoplasm chemotherapy

Search 2:

AMH cancer ovarian reserve

Search 3:

"anti-Mullerian hormone" breast cancer/neoplasm

Search 4:

"anti-Mullerian hormone" lymphoma

Search 5:

"anti-Mullerian hormone" thyroid cancer/neoplasm radioiodine

Search 6:

"anti-Mullerian hormone" childhood cancer/neoplasm survivor

Search 7:

AMH adolescent young adult cancer/neoplasm

**Supplementary Table S1**. Risk of bias appraisal

| **Study** | **Year** | **Title** | **Analysis stratum for this review** | **Selection bias** | **Follow-up / attrition bias** | **Outcome measurement bias** | **Confounding / co-intervention bias** | **Reporting bias** | **Overall ROB** |
| --- | --- | --- | --- | --- | --- | --- | --- | --- | --- |
| Anderson RA | 2011 | Pretreatment serum anti-Müllerian hormone predicts long-term ovarian function and bone mass after chemotherapy for early breast cancer. J Clin Endocrinol Metab 96:1336–1343. | Primary pre/post synthesis | **Moderate** | **Moderate** | **Low** | **Moderate** | **Moderate** | **Moderate** |
| Anderson RA | 2017 | The utility of anti-Müllerian hormone in the diagnosis and prediction of loss of ovarian function following chemotherapy for early breast cancer | Primary pre/post synthesis | **Moderate** | **Moderate** | **Low** | **Moderate** | **Moderate** | **Moderate** |
| Anderson RA | 2018 | Determinants of ovarian function after response-adapted therapy in patients with advanced Hodgkin's lymphoma (RATHL): a secondary analysis of a randomised phase 3 trial | Primary pre/post synthesis | **Low** | **Moderate** | **Low** | **Moderate** | **Moderate** | **Moderate** |
| Anderson RA | 2022 | Diagnostic and predictive accuracy of anti-mullerian hormone for ovarian function after chemotherapy in premenopausal women with early breast cancer | Primary pre/post synthesis | **Moderate** | **Moderate** | **Low** | **Moderate** | **Moderate** | **Moderate** |
| Bath LE | 2003 | Depletion of ovarian reserve in young women after treatment for cancer in childhood: detection by anti‐Müllerian hormone, inhibin B and ovarian ultrasound | Systematic-review only; not in quantitative synthesis | **Moderate** | **Moderate** | **Low** | **Moderate** | **Low** | **Moderate** |
| Behringer K | 2013 | Gonadal function and fertility in survivors after Hodgkin lymphoma treatment within the German Hodgkin Study Group HD13 to HD15 trials. J Clin Oncol 31:231–239. | Longitudinal / post-treatment only | **High** | **Moderate** | **Moderate** | **High** | **Moderate** | **High** |
| Ben-Aharon I | 2015 | Long-term follow-up of chemotherapy-induced ovarian failure in young breast cancer patients: the role of vascular toxicity | Primary pre/post synthesis | **Moderate** | **Moderate** | **Low** | **Moderate** | **Moderate** | **Moderate** |
| Berjeb KK | 2021 | Evaluation of ovarian reserve before and after chemotherapy. J Gynecol Obstet Hum Reprod 50:102035. | Primary pre/post synthesis | **Moderate** | **Moderate** | **Low** | **Moderate** | **Moderate** | **Moderate** |
| Brougham MFH | 2012 | Anti-Müllerian hormone is a marker of gonadotoxicity in pre- and postpubertal girls treated for cancer: a prospective study. | Primary pre/post synthesis | **Moderate** | **Moderate** | **Low** | **Moderate** | **Moderate** | **Moderate** |
| Cameron K | 2019 | Differential rates of change in measures of ovarian reserve in young cancer survivors across the reproductive lifespan. J Clin Endocrinol Metab 104:1813–1822. | Longitudinal / post-treatment only | **High** | **Moderate** | **Moderate** | **High** | **Moderate** | **High** |
| Charpentier A-M | 2014 | Anti-Müllerian hormone screening to assess ovarian reserve among female survivors of childhood cancer. | Longitudinal / post-treatment only | **High** | **Moderate** | **Moderate** | **High** | **Moderate** | **High** |
| Chemerinski A | 2020 | elationship of menopausal symptoms and ovarian reserve in reproductive-aged cancer survivors. | Systematic-review only; not in quantitative synthesis | **Moderate** | **High** | **Moderate** | **Moderate** | **Low** | **Moderate** |
| Decanter C | 2010 | Anti-Müllerian hormone follow-up in young women treated by chemotherapy for lymphoma: preliminary results | Primary pre/post synthesis | **Moderate** | **Moderate** | **Low** | **Moderate** | **Moderate** | **Moderate** |
| Decanter C | 2018 | Different patterns of ovarian recovery after cancer treatment suggest various individual ovarian susceptibilities to chemotherapy | Primary pre/post synthesis | **High** | **Moderate** | **Moderate** | **High** | **Moderate** | **High** |
| Decanter C | 2021 | Longitudinal study of AMH variations in 122 adolescents and young adults (AYA) and non-AYA lymphoma patients to evaluate the chemo-induced ovarian toxicity to further personalise fertility preservation counselling | Primary pre/post synthesis; Systematic-review only; not in quantitative synthesis | **Moderate** | **Moderate** | **Low** | **Moderate** | **Low** | **Moderate** |
| Demeestere I | 2016 | No evidence for the benefit of gonadotropin-releasing hormone agonist in preserving ovarian function and fertility in lymphoma survivors treated with chemotherapy: final long-term report of a prospective randomized trial. J Clin Oncol 34:2568–2574. | Longitudinal / post-treatment only | **Low** | **Moderate** | **Low** | **Low** | **Moderate** | **Moderate** |
| Demeestere I | 2021 | Gonadal function recovery in patients with advanced Hodgkin lymphoma treated with a PET-adapted regimen: prospective analysis of a randomized phase III trial (AHL2011). J Clin Oncol 39:3251–3260. | Primary pre/post synthesis | **Low** | **Moderate** | **Low** | **Moderate** | **Low** | **Moderate** |
| Dezellus A | 2017 | Prospective evaluation of serum anti-Müllerian hormone dynamics in 250 women of reproductive age treated with chemotherapy for breast cancer | Primary pre/post synthesis | **Moderate** | **Moderate** | **Low** | **Moderate** | **Moderate** | **Moderate** |
| Di Paola R | 2013 | Anti-Müllerian hormone and antral follicle count reveal a late impairment of ovarian reserve in patients undergoing low-gonadotoxic regimens for hematological malignancies | Longitudinal / post-treatment only | **Moderate** | **Moderate** | **Low** | **Moderate** | **Low** | **Moderate** |
| Dillon KE | 2012 | Pre-Treatment AMH Determines Rate of Post-Therapy Ovarian Reserve Recovery: Acute Changes in Ovarian Reserve During and After Chemotherapy | Systematic-review only; not in quantitative synthesis | **Moderate** | **Moderate** | **Low** | **Moderate** | **Low** | **Moderate** |
| Dillon KE | 2013 | Pregnancy after cancer: results from a prospective cohort study of cancer survivors | Longitudinal / post-treatment only | **Moderate** | **Moderate** | **Low** | **High** | **Moderate** | **High** |
| Drechsel KCE | 2024 | The impact of treatment for childhood classical Hodgkin lymphoma according to the EuroNet-PHL-C2 protocol on serum anti-Müllerian hormone | Primary pre/post synthesis | **Moderate** | **Moderate** | **Low** | **Moderate** | **Moderate** | **Moderate** |
| Elchuri SV | 2016 | Low anti-Müllerian hormone in pediatric cancer survivors in the early years after gonadotoxic therapy. | Longitudinal / post-treatment only | **High** | **Moderate** | **Moderate** | **High** | **Moderate** | **High** |
| Elgindy EA | 2013 | Gonadotrophin suppression to prevent chemotherapy-induced ovarian damage: a randomized controlled trial | Primary pre/post synthesis | **Low** | **Moderate** | **Low** | **Moderate** | **Moderate** | **Moderate** |
| Elitzur S | 2021 | Long-term ovarian reserve and fertility outcomes in female survivors of childhood acute lymphoblastic leukemia | Longitudinal / post-treatment only | **High** | **Moderate** | **Moderate** | **High** | **Moderate** | **High** |
| Evranos B | 2018 | Effects of radioactive iodine therapy on ovarian reserve: a prospective pilot study | Primary pre/post synthesis | **High** | **High** | **Low** | **Moderate** | **Moderate** | **High** |
| George SA | 2019 | Assessment of ovarian function in adolescents and young adults after childhood cancer treatment—How accurate are young adult/parent proxy-reported outcomes? | Systematic-review only; not in quantitative synthesis | **High** | **High** | **Moderate** | **High** | **Moderate** | **High** |
| Gharwan H | 2016 | Female fertility following dose-adjusted EPOCH-R chemotherapy in primary mediastinal B-cell lymphomas | Primary pre/post synthesis | **Moderate** | **Moderate** | **Low** | **Moderate** | **Moderate** | **Moderate** |
| Goldfarb SB | 2021 | Impact of adjuvant chemotherapy or tamoxifen alone on the ovarian reserve of young women with breast cancer | Primary pre/post synthesis | **Moderate** | **Moderate** | **Low** | **Moderate** | **Moderate** | **Moderate** |
| Gracia CR | 2012 | Impact of cancer therapies on ovarian reserve | Systematic-review only; not in quantitative synthesis | **Low** | **Moderate** | **Low** | **Low** | **Moderate** | **Moderate** |
| Gupta AA | 2016 | Anti-Müllerian hormone in female adolescent cancer patients before, during, and after completion of therapy: a pilot feasibility study. . | Primary pre/post synthesis | **High** | **High** | **Low** | **Moderate** | **Moderate** | **High** |
| Hamre H | 2012 | Gonadal function and parenthood 20 years after treatment for childhood lymphoma: a cross-sectional study | Longitudinal / post-treatment only | **High** | **High** | **Moderate** | **High** | **Moderate** | **High** |
| Hamy A-S | 2016 | Anti-Müllerian hormone in breast cancer patients treated with chemotherapy: a retrospective evaluation of subsequent pregnancies | Primary pre/post synthesis; Longitudinal / post-treatment only | **High** | **High** | **Low** | **Moderate** | **Moderate** | **High** |
| Henry NL | 2014 | Prediction of postchemotherapy ovarian function using markers of ovarian reserve | Primary pre/post synthesis | **Moderate** | **Moderate** | **Low** | **Moderate** | **Moderate** | **Moderate** |
| Jin HY | 2023 | Gonadal function in female adolescent and young adult survivors of childhood cancer | Longitudinal / post-treatment only | **High** | **Moderate** | **Moderate** | **High** | **Moderate** | **High** |
| Jingrukwong L | 2025 | Assessing the ovarian reserve in girls who survived childhood leukemia using anti-Müllerian hormone | Longitudinal / post-treatment only | **High** | **Moderate** | **Moderate** | **High** | **Moderate** | **High** |
| Kim HA | 2018 | Post-chemotherapy serum anti-Müllerian hormone level predicts ovarian function recovery | Longitudinal / post-treatment only | **Moderate** | **Moderate** | **Low** | **Moderate** | **Moderate** | **Moderate** |
| Krawczuk-Rybak M | 2013a | Anti-Müllerian hormone as a sensitive marker of ovarian function in young cancer survivors | Longitudinal / post-treatment only | **High** | **Moderate** | **Moderate** | **High** | **Moderate** | **High** |
| Krawczuk-Rybak M | 2013b | The progressive reduction in the ovarian reserve in young women after anticancer treatment | Longitudinal / post-treatment only | **High** | **Moderate** | **Moderate** | **High** | **Moderate** | **High** |
| Krawczuk-Rybak M | 2019 | The influence of different intensity of treatment on hormonal markers of gonadal function in acute lymphoblastic leukemia survivors | Longitudinal / post-treatment only | **High** | **Moderate** | **Moderate** | **High** | **Moderate** | **High** |
| Lambertini M | 2019 | Impact of taxanes, endocrine therapy, and deleterious germline BRCA mutations on anti-Müllerian hormone levels in early breast cancer patients treated with anthracycline- and cyclophosphamide-based chemotherapy | Primary pre/post synthesis; Longitudinal / post-treatment only | **Moderate** | **Moderate** | **Low** | **Moderate** | **Moderate** | **Moderate** |
| Lambertini M | 2025 | Predicting ovarian function loss after chemotherapy and anti-HER2 therapy in young breast cancer patients. J Natl Cancer Inst (in press). | Primary pre/post synthesis | **Moderate** | **Moderate** | **Low** | **Moderate** | **Moderate** | **Moderate** |
| Lee D-Y | 2018 | Prediction of ovarian function recovery in young breast cancer patients after protection with gonadotropin-releasing hormone agonist during chemotherapy | Longitudinal / post-treatment only | **Moderate** | **Moderate** | **Low** | **Moderate** | **Moderate** | **Moderate** |
| Lee DY | 2020 | Prediction of successful ovarian protection using gonadotropin-releasing hormone agonists during chemotherapy in young estrogen receptor-negative breast cancer patients | Primary pre/post synthesis | **Moderate** | **Moderate** | **Low** | **Moderate** | **Moderate** | **Moderate** |
| Leiper A | 2020 | Anti-Müllerian hormone and Inhibin B after stem cell transplant in childhood: a comparison of myeloablative, reduced intensity and treosulfan-based chemotherapy regimens. | Systematic-review only; not in quantitative synthesis | **Moderate** | **Moderate** | **Low** | **Moderate** | **Low** | **Moderate** |
| Leonard RCF | 2017 | GnRH agonist for protection against ovarian toxicity during chemotherapy for early breast cancer: the Anglo Celtic Group OPTION trial | Primary pre/post synthesis; Longitudinal / post-treatment only | **Low** | **Moderate** | **Low** | **Low** | **Moderate** | **Moderate** |
| Li X | 2020 | Can anti-Müllerian hormone be a reliable biomarker for assessing ovarian function in women postchemotherapy?. | Primary pre/post synthesis | **Moderate** | **Low** | **Moderate** | **Moderate** | **Moderate** | **Moderate** |
| Lie Fong S | 2009 | Assessment of ovarian reserve in adult childhood cancer survivors using anti-Müllerian hormone | Longitudinal / post-treatment only | **High** | **Moderate** | **Moderate** | **High** | **Moderate** | **High** |
| Loubersac S | 2020 | Evolution of serum anti-Müllerian hormone level in young women treated with chemotherapy for breast cancer according to basal AMH level | Longitudinal / post-treatment only | **Moderate** | **Moderate** | **Low** | **Moderate** | **Moderate** | **Moderate** |
| Lunsford AJ | 2014 | Anti-Müllerian hormone as a measure of reproductive function in female childhood cancer survivors | Longitudinal / post-treatment only | **High** | **Moderate** | **Moderate** | **High** | **Moderate** | **High** |
| Lutchman Singh K | 2007 | Predictors of ovarian reserve in young women with breast cancer | Primary pre/post synthesis | **High** | **Moderate** | **Moderate** | **High** | **Moderate** | **High** |
| Malisic E | 2018 | Assessment of ovarian function after chemotherapy in women with early and locally advanced breast cancer from Serbia | Longitudinal / post-treatment only | **Moderate** | **Moderate** | **Low** | **Moderate** | **Moderate** | **Moderate** |
| Mittica M | 2020 | Cross-sectional and prospective study on anti-Müllerian hormone changes in a cohort of pre-menopausal women with a history of differentiated thyroid cancer | Primary pre/post synthesis | **Moderate** | **Moderate** | **Low** | **High** | **Moderate** | **High** |
| Miyoshi Y | 2012 | Low serum concentrations of anti-Müllerian hormone are common in 53 female childhood cancer survivors | Longitudinal / post-treatment only | **High** | **Moderate** | **Moderate** | **High** | **Moderate** | **High** |
| Morarji K | 2017 | Ovarian function after chemotherapy in young breast cancer survivors | Longitudinal / post-treatment only | **High** | **Moderate** | **Moderate** | **High** | **Moderate** | **High** |
| Mörse H | 2016 | Severe gonadotoxic insult manifests early in young girls treated for Ewing sarcoma | Primary pre/post synthesis | **Moderate** | **Moderate** | **Low** | **Moderate** | **Moderate** | **Moderate** |
| Nielsen SN | 2013 | A 10-year follow up of reproductive function in women treated for childhood cancer | Longitudinal / post-treatment only | **Moderate** | **Moderate** | **Low** | **High** | **Moderate** | **High** |
| Nies M | 2020 | Long-term effects of radioiodine treatment on female fertility in survivors of childhood differentiated thyroid carcinoma | Longitudinal / post-treatment only | **High** | **Moderate** | **Moderate** | **High** | **Moderate** | **High** |
| Nilsson S | 2022 | A survey of ovarian reserve and quality of life in female survivors of pediatric cancer | Longitudinal / post-treatment only | **High** | **High** | **Moderate** | **High** | **Moderate** | **High** |
| Nitzschke M | 2010 | GnRH analogs do not protect ovaries from chemotherapy-induced ultrastructural injury in Hodgkin’s lymphoma patients | Longitudinal / post-treatment only | **High** | **Moderate** | **Moderate** | **High** | **Moderate** | **High** |
| Nyström A | 2019 | Anti-müllerian hormone compared with other ovarian markers after childhood cancer treatment | Longitudinal / post-treatment only | **High** | **Moderate** | **Moderate** | **High** | **Moderate** | **High** |
| Palinska-Rudzka KE | 2019 | Five-year study assessing the clinical utility of anti-Müllerian hormone measurements in reproductive-age women with cancer | Primary pre/post synthesis; Longitudinal / post-treatment only | **High** | **Moderate** | **Moderate** | **High** | **Moderate** | **High** |
| Parissone F | 2021 | Female adolescents and young women previously treated for pediatric malignancies: assessment of ovarian reserve and gonadotoxicity risk stratification for early identification of patients at increased infertility risk. | Longitudinal / post-treatment only | **High** | **Moderate** | **Moderate** | **High** | **Moderate** | **High** |
| Roshandel R | 2021 | Female reproductive function after treatment of childhood acute lymphoblastic leukemia | Longitudinal / post-treatment only | **High** | **Moderate** | **Moderate** | **High** | **Moderate** | **High** |
| Shandley LM | 2017 | Impact of tamoxifen therapy on fertility in breast cancer survivors | Longitudinal / post-treatment only | **High** | **Moderate** | **Moderate** | **High** | **Moderate** | **High** |
| Signorino C | 2025 | Ovarian function in adolescents and young adults undergoing cancer treatment: biochemical and ultrasound marker analysis | Longitudinal / post-treatment only | **Moderate** | **Moderate** | **Low** | **Moderate** | **Moderate** | **Moderate** |
| Silva C | 2019 | Adverse reproductive health outcomes in a cohort of young women with breast cancer exposed to systemic treatments. J Ovarian Res 12:102. | Primary pre/post synthesis | **High** | **Moderate** | **Moderate** | **High** | **Moderate** | **High** |
| Singh MP | 2025 | Age and serum anti-Müllerian hormone levels as predictors of time to return of menses after chemotherapy | Primary pre/post synthesis | **Moderate** | **Moderate** | **Low** | **Moderate** | **Moderate** | **Moderate** |
| Su HI | 2010 | Antimullerian hormone and inhibin B are hormone measures of ovarian function in late reproductive-aged breast cancer survivors | Longitudinal / post-treatment only | **High** | **Moderate** | **Moderate** | **High** | **Moderate** | **High** |
| Su HI | 2020 | Modeling variation in the reproductive lifespan of female adolescent and young adult cancer survivors using AMH. J Clin Endocrinol Metab 105:2740–2751. | Longitudinal / post-treatment only | **High** | **Moderate** | **Moderate** | **High** | **Moderate** | **High** |
| Thomas-Teinturier C | 2015 | Ovarian reserve after treatment with alkylating agents during childhood | Longitudinal / post-treatment only | **High** | **Moderate** | **Moderate** | **High** | **Moderate** | **High** |
| Trapp E | 2017 | Anti-Müllerian hormone (AMH) levels in premenopausal breast cancer patients treated with taxane-based adjuvant chemotherapy: a translational research project of the SUCCESS A study | Primary pre/post synthesis | **Moderate** | **Moderate** | **Low** | **Moderate** | **Moderate** | **Moderate** |
| van Beek RD | 2007 | Anti-Müllerian hormone is a sensitive serum marker for gonadal function in women treated for Hodgkin’s lymphoma during childhood. J Clin Endocrinol Metab 92:3869–3874. | Longitudinal / post-treatment only | **High** | **Moderate** | **Moderate** | **High** | **Low** | **High** |
| van der Kooi AL | 2017 | Longitudinal follow-up in female childhood cancer survivors: no signs of accelerated ovarian function loss | Longitudinal / post-treatment only | **Moderate** | **Moderate** | **Low** | **High** | **Moderate** | **High** |
| van Velsen EFS | 2020 | Longitudinal analysis of the effect of radioiodine therapy on ovarian reserve in females with differentiated thyroid cancer | Primary pre/post synthesis | **Moderate** | **Moderate** | **Low** | **Moderate** | **Moderate** | **Moderate** |
| Wenners A | 2017 | Reduced ovarian reserve in young early breast cancer patients: preliminary data from a prospective cohort trial | Primary pre/post synthesis; Longitudinal / post-treatment only | **High** | **High** | **Low** | **Moderate** | **Moderate** | **High** |
| Yaish I | 2018 | A single radioactive iodine treatment has a deleterious effect on ovarian reserve in women with thyroid cancer: results of a prospective pilot study | Primary pre/post synthesis | **High** | **High** | **Low** | **Moderate** | **Moderate** | **High** |
| Yu B | 2010 | Changes in markers of ovarian reserve and endocrine function in young women with breast cancer undergoing adjuvant chemotherapy | Primary pre/post synthesis | **Moderate** | **Moderate** | **Low** | **Moderate** | **Moderate** | **Moderate** |
| Zhong Y | 2019 | GnRHa for ovarian protection and the association between AMH and ovarian function during adjuvant chemotherapy for breast cancer. J Cancer 10:4278–4285. | Primary pre/post synthesis | **Moderate** | **Moderate** | **Low** | **Moderate** | **Moderate** | **Moderate** |

**Supplementary Figure S1: Risk of bias summary figure**

**Supplementary Table S2. Crude data per graph**

| Cumulative_Breast cancer | | | |  |  |  |
| --- | --- | --- | --- | --- | --- | --- |
| Months | Mean | SD | N |  |  |  |
| 0 | 2.15 | 0.7475 | 2440 |  |  |  |
| 6 | 0.13678 | 0.0305 | 1635 |  |  |  |
| 12 | 0.39 | 0.105 | 1601 |  |  |  |
| 18 | 0.302306 | 0.2 | 737 |  |  |  |
| 24 | 0.4 | 0.286487 | 755 |  |  |  |
|  |  |  |  |  |  |  |
| Cumulative_non-ABVD-AVD | | | |  |  |  |
| Months | Mean | SD | N |  |  |  |
| 0 | 2.337261 | 0.2 | 159 |  |  |  |
| 6 | 0.297942 | 0.01 | 159 |  |  |  |
| 12 | 0.365019 | 0.01 | 159 |  |  |  |
| 18 | 1.02 | 0.01 | 159 |  |  |  |
| 24 | 0.3525 | 0.01 | 159 |  |  |  |
|  |  |  |  |  |  |  |
| Cumulative_ABVD-AVD | | | |  |  |  |
| Months | Mean | SD | N |  |  |  |
| 0 | 1.97 | 0.25 | 187 |  |  |  |
| 6 | 0.6912 | 0.02 | 187 |  |  |  |
| 12 | 1.241667 | 0.02 | 187 |  |  |  |
| 18 | 0.415 | 0.02 | 187 |  |  |  |
| 24 | 0.806667 | 0.03 | 187 |  |  |  |
|  |  |  |  |  |  |  |
|  | Cumulative_Thyroid | | |  |  |  |
| Months | Mean | SD | N |  |  |  |
| 0 | 3.25 | 0.8 | 201 |  |  |  |
| 6 | 1.125 | 0.55 | 92 |  |  |  |
| 12 | 1.25 | 0.35 | 201 |  |  |  |
| 18 |  |  |  |  |  |  |
| 24 | 1.22 | 1.19 | 85 |  |  |  |
| 30 |  |  |  |  |  |  |
| 36 | 1.07 | 1.21 | 85 |  |  |  |
| 42 |  |  |  |  |  |  |
| 48 | 0.95 | 1.23 | 85 |  |  |  |
|  |  |  |  |  |  |  |
| Longitudinal_Adult | | | | | | |
| Years | Adult_Gonadotoxicity | | | Adult_Control | | |
| 1 | 0.355 | 0.399845 | 2698 | 1.41 | 0.4 | 700 |
| 2 | 0.67 | 0.484914 | 2698 | 1.26 | 0.44 | 700 |
| 3 | 0.613125 | 0.458637 | 2698 | 1.1 | 0.43 | 700 |
| 4 | 0.688125 | 0.673661 | 2698 | 1.08 | 0.4 | 700 |
| 5 | 1.165 | 0.471471 | 2698 | 1.02 | 0.5 | 700 |
| 6 | 1.47211 | 0.550234 | 2698 | 1 | 0.34 | 700 |
| 7 | 1.444133 | 0.444133 | 2698 | 0.95 | 0.4 | 700 |
| 8 | 1.514446 | 0.373821 | 2698 | 0.96 | 0.5 | 700 |
| 9 | 1.069753 | 0.329128 | 2698 | 0.92 | 0.3 | 700 |
| 10 | 1.380368 | 0.239743 | 2698 | 0.9 | 0.4 | 700 |
| 11 | 1.335677 | 1.195052 | 2698 | 0.95 | 0.44 | 700 |
| 12 | 0.803953 | 0.282078 | 2698 | 0.9 | 0.43 | 700 |
| 13 | 1.097852 | 0.254102 | 2698 | 0.85 | 0.4 | 700 |
| 14 | 0.96875 | 0.181159 | 2698 | 0.85 | 0.5 | 700 |
| 15 | 1.151275 | 0.932524 | 2698 | 0.89 | 0.34 | 700 |
| 16 | 1.029547 | 0.920172 | 2698 | 0.83 | 0.4 | 700 |
| 17 | 0.858767 | 1.046267 | 2698 | 0.79 | 0.5 | 700 |
| 18 | 0.696883 | 1.118758 | 2698 | 0.72 | 0.3 | 700 |
| 19 | 0.633117 | 0.914366 | 2698 | 0.68 | 0.4 | 700 |
| 20 | 0.673271 | 0.829521 | 2698 | 0.65 | 0.44 | 700 |
| 21 | 0.5103 | 0.9478 | 2698 | 0.65 | 0.43 | 700 |
| 22 | 0.328125 | 0 | 2698 | 0.5 | 0.4 | 700 |
| 23 | 0.09375 | 0 | 2698 | 0.41 | 0.5 | 700 |
| 24 | 0.171875 | 0 | 2698 | 0.31 | 0.34 | 700 |
| 25 | 0.234375 | 0 | 2698 | 0.25 | 0.4 | 700 |
|  |  |  |  |  |  |  |
| Longitudinal_Paediatric | | | | | | |
| Years | Paediatric_Gonadotoxicity | | | Paediatric_Control | | |
| 1 | 0.22 | 0.2 | 1649 | 2.67 | 0.8 | 800 |
| 2 | 0.74 | 0.03 | 1649 | 2.73 | 0.87 | 800 |
| 3 | 1.27 | 0.12 | 1649 | 2.84 | 0.87 | 800 |
| 4 | 1.31 | 0.1 | 1649 | 2.93 | 0.9 | 800 |
| 5 | 2.075 | 0.1 | 1649 | 3.01 | 0.65 | 800 |
| 6 | 1.6 | 0.1 | 1649 | 3.14 | 0.7 | 800 |
| 7 | 1.07 | 0.1 | 1649 | 3.4 | 0.55 | 800 |
| 8 | 2.35 | 0.73 | 1649 | 3.27 | 0.8 | 800 |
| 9 | 2.23 | 0.1 | 1649 | 3.52 | 0.87 | 800 |
| 10 | 2.14 | 0.32 | 1649 | 3.64 | 0.9 | 800 |
| 11 | 2.445 | 1.159684 | 1649 | 3.8 | 0.65 | 800 |
| 12 | 2.5451 | 0.4 | 1649 | 3.75 | 0.8 | 800 |
| 13 | 2.2507 | 0.4293 | 1649 | 3.65 | 0.87 | 800 |
| 14 | 2.9756 | 0 | 1649 | 3.6 | 0.9 | 800 |
| 15 | 2.2554 | 0.5554 | 1649 | 3.5 | 0.8 | 800 |
| 16 | 2.9906 | 0.4 | 1649 | 3.4 | 0.87 | 800 |
| 17 | 3.036996 | 0.898596 | 1649 | 3.3 | 0.9 | 800 |
| 18 | 3.02507 | 0.76137 | 1649 | 3.2 | 0.65 | 800 |
| 19 | 3.954057 | 0.574757 | 1649 | 3.06 | 0.8 | 800 |
| 20 | 3.5078 | 0 | 1649 | 2.77 | 0.87 | 800 |
| 21 | 2.05205 | 0.05205 | 1649 | 2.5 | 0.8 | 800 |
| 22 | 2.2454 | 0.424322 | 1649 | 2.16 | 0.65 | 800 |
| 23 | 2.3771 | 0 | 1649 | 1.97 | 0.87 | 800 |
| 24 | 1.5281 | 0 | 1649 | 1.79 | 0.8 | 800 |
| 25 | 1.48955 | 0.18955 | 1649 | 1.83 | 0.87 | 800 |

**Supplementary Table S3. Mixed population studies or those that did not offer extractable data for inclusion in the longitudinal cohorts but were included in the systematic review.**

| **Publication** | **Study design** | **Mixed (Median Age, range)/Adult or Paediatric** | **Cohort size** | **Cancer type** | **AMH assay** | **Time since diagnosis** |
| --- | --- | --- | --- | --- | --- | --- |
| [Bath LE, et al. 2003](https://academic.oup.com/humrep/article/18/11/2368/645226?login=true) | Prospective cross-sectional | 24 (16–34) | 20 | ALL, Ewings, Medulloblastoma, Rhabdomyosarcoma, Wilms, lymphoma, osteosarcoma | ELISA (Beckman Coulter | > 2 years |
| [Chemerinski A, et al. 2020](https://link.springer.com/article/10.1007/s11764-020-00857-z) | Prospective longitudinal | 25 (15–39) | 171 | Lymphoma, leukaemia, sarcoma, breast, Wilm's and other | picoAMH ELISA (Ansh Labs) | mean 3.1 years (range 1.1–11.3 years) |
| [Decanter C, et al. 2021](https://academic.oup.com/humrep/article/36/10/2743/6356123?login=true) | Prospective longitudinal | 24.8 (17–32.3) | 122 | Lymphoma | Gen II ELISA (Beckman Coulter) | 24 months |
| [Gracia CR, et al. 2012](https://pmc.ncbi.nlm.nih.gov/articles/PMC4005036/) | Retrospective longitudinal | 26.4 (15-39) | 71 | Lymphoma, leukaemia, sarcoma, Wilm's tumour, Breast and other | ELISA (Diagnostic Systems Laboratories) | 14 years |
| [Leiper A, et al. 2020](https://www.nature.com/articles/s41409-020-0866-9) | Prospective longitudinal and cross-sectional | 20.93 (7.3-26) | 121 | Mixed, all treated with HSCT | Gen II ELISA (Beckman Coulter) | 9.89 (±5.63) [range 1.8–23.8] years |
| Dillon et al., 2012 | Prospective longitudinal | 26.1 (15.0–35.9) | 46 | Breast (19/46), Leukemia (9/46), Lymphoma (13/46), Sarcoma (4/46), Brain (1/46), Wilm’s (1/46), Germ cell (1/46), Other (2/46) | AMH ELISA (Diagnostic Systems) | 9 months |
| [George SA, et al. 2019](https://www.liebertpub.com/doi/abs/10.1089/jayao.2018.0080?journalCode=jayao) | Prospective cross-sectional | Paediatric | 190 | Leukemia, HL, neuroblastoma, osteosarcoma, other sarcoma, Wilm's tumour and other | Multiple assays | 7.3 (4.1) years |

AMH: anti-Müllerian hormone; ELISA, Enzyme-Linked Immunosorbent Assay;

**Supplementary Data File S2. Supplementary references**

Anderson RA, Cameron DA. Pretreatment serum anti-Müllerian hormone predicts long-term ovarian function and bone mass after chemotherapy for early breast cancer. *J Clin Endocrinol Metab* 2011;**96:**1336–1343.

Anderson RA, Mansi J, Coleman RE, Adamson DJA, Leonard RCF. The utility of anti-Müllerian hormone in the diagnosis and prediction of loss of ovarian function following chemotherapy for early breast cancer. *Eur J Cancer* 2017;**87:**58–64.

Anderson RA, Lambertini M, Hall PS, Wallace WH, Morrison DS, Kelsey TW. Survival after breast cancer in women with a subsequent live birth: influence of age at diagnosis and interval to subsequent pregnancy. *Eur J Cancer* 2022;**173:**113–122.

Anderson RA, Wahab H, Zhong X, Welt CK, Finkelstein JS, Peppercorn J, Partridge AH. Determinants of ovarian function after response-adapted therapy in patients with advanced Hodgkin's lymphoma (RATHL): a secondary analysis of a randomised phase 3 trial. *Lancet Haematol* 2018;**5:**e522–e530.

Bath LE, Wallace WH, Shaw MP, Fitzpatrick C, Anderson RA. Depletion of ovarian reserve in young women after treatment for cancer in childhood: detection by anti-Müllerian hormone, inhibin B and ovarian ultrasound. *Hum Reprod* 2003;**18:**2368–2374.

Behringer K, Thielen I, Mueller H, Goergen H, Smolka V, Kreissl S, Reiners KS, Diehl V, Engert A, Borchmann P. Gonadal function and fertility in survivors after Hodgkin lymphoma treatment within the German Hodgkin Study Group HD13 to HD15 trials. *J Clin Oncol* 2013;**31:**231–239.

Ben-Aharon I, Bar-Joseph H, Tzarfaty G, Kuchinsky L, Rizel S, Stemmer SM, Shalgi R. Long-term follow-up of chemotherapy-induced ovarian failure in young breast cancer patients: the role of vascular toxicity. *Oncologist* 2015;**20:**985–991.

Berjeb KK, Kaabia O, Mourali M, Kehila M, Faleh R, Mahjoub T. Evaluation of ovarian reserve before and after chemotherapy. *J Gynecol Obstet Hum Reprod* 2021;**50:**102035.

Brougham MFH, Crofton PM, Johnson EJ, Evans N, Anderson RA, Wallace WHB. Anti-Müllerian hormone is a marker of gonadotoxicity in pre- and postpubertal girls treated for cancer: a prospective study. *J Clin Endocrinol Metab* 2012;**97:**2059–2067.

Cameron K, Su HI, Bhala S, Gracia CR, Sammel MD. Differential rates of change in measures of ovarian reserve in young cancer survivors across the reproductive lifespan. *J Clin Endocrinol Metab* 2019;**104:**1813–1822.

Charpentier A-M, Barton S, Mercier C, Labidi-Galy I, Neven P, Riedel C, Casasnovas O, Lambertini M. Anti-Müllerian hormone screening to assess ovarian reserve among female survivors of childhood cancer. *Pediatr Blood Cancer* 2014;**61:**1807–1812.

Chemerinski A, Bharat S, Odell SG, Kim JG, Silber SJ, Sammel MD, Gracia CR. Longitudinal anti-Müllerian hormone levels in women with cancer before and after treatment. *J Cancer Surviv* 2020;**14:**883–891.

Decanter C, Cloquet M, Pigny P, Mery E, Lefebvre C, Dewailly D. Different patterns of ovarian recovery after cancer treatment suggest various individual ovarian susceptibilities to chemotherapy. *J Assist Reprod Genet* 2018;**35:**423–430.

Decanter C, Morschhauser F, Pigny P, Lefebvre C, Gallo C, Dewailly D. Anti-Müllerian hormone follow-up in young women treated by chemotherapy for lymphoma: preliminary results. *Reprod Biomed Online* 2010;**20:**280–285.

Decanter C, Pigny P, Lefebvre C, Cantin J, Catteau-Jonard S, Morschhauser F, Dewailly D. Longitudinal study of AMH variations in 122 adolescents and young adults (AYA) and non-AYA lymphoma patients to evaluate the chemo-induced ovarian toxicity to further personalise fertility preservation counselling. *Hum Reprod* 2021;**36:**2743–2753.

Demeestere I, Brice P, Peccatori FA, Kentos A, Dupuis J, Zachee P, Casasnovas O, Van Den Neste E, Doyen C, Van Eygen K et al. No evidence for the benefit of gonadotropin-releasing hormone agonist in preserving ovarian function and fertility in lymphoma survivors treated with chemotherapy: final long-term report of a prospective randomized trial. *J Clin Oncol* 2016;**34:**2568–2574.

Demeestere I, Tajahmady A, Brice P, Bordessoule D, Ferme C, Casasnovas RO, Morschhauser F, Ghesquieres H, Jardin F, Bonnet C et al. Gonadal function recovery in patients with advanced Hodgkin lymphoma treated with a PET-adapted regimen: prospective analysis of a randomized phase III trial (AHL2011). *J Clin Oncol* 2021;**39:**3251–3260.

Dezellus A, Barriere P, Campone M, Comete M, Fréour T. Prospective evaluation of serum anti-Müllerian hormone dynamics in 250 women of reproductive age treated with chemotherapy for breast cancer. *Eur J Cancer* 2017;**79:**72–80.

Di Paola R, Costantini C, Tecchio C, Salvagno GL, Montemezzi R, Perandini A, Tosi F, Franchi M, Moghetti P. Anti-Müllerian hormone and antral follicle count reveal a late impairment of ovarian reserve in patients undergoing low-gonadotoxic regimens for hematological malignancies. *Oncologist* 2013;**18:**1307–1314.

Dillon KE, Sammel MD, Prewitt M, Ginsberg JP, Walker D, Mersereau JE, Gosiengfiao Y, Gracia CR. Pre-treatment AMH determines rate of post-therapy ovarian reserve recovery: acute changes in ovarian reserve during and after chemotherapy. *Fertil Steril* 2012;**99:**477–483.

Dillon KE, Sammel MD, Prewitt M, Ginsberg JP, Walker D, Mersereau JE, Gosiengfiao Y, Gracia CR. Pregnancy after cancer: results from a prospective cohort study of cancer survivors. *Pediatr Blood Cancer* 2013;**60:**2001–2007.

Drechsel KCE, Borgmann-Staudt A, Bidlingmaier M, Dokoupil K, Escherich G, Schoppe M, Haas R, Mann G, Schmid I, Suttorp M et al. The impact of treatment for childhood classical Hodgkin lymphoma according to the EuroNet-PHL-C2 protocol on serum anti-Müllerian hormone. *Br J Haematol* 2024;**39:**1701–1711.

Elchuri SV, Bhakta N, Howell CR, Partin RE, Chemaitilly W, Sklar CA, Hudson MM, Robison LL, Ness KK, Srivastava DK et al. Low anti-Müllerian hormone in pediatric cancer survivors in the early years after gonadotoxic therapy. *J Adolesc Young Adult Oncol* 2016;**5:**150–158.

Elgindy EA, El-Haieg DO, Khorshid OM, Ismail EI, Abdelgawad M, Sallam HN, Abou-Setta AM. Gonadotrophin suppression to prevent chemotherapy-induced ovarian damage: a randomized controlled trial. *Obstet Gynecol* 2013;**121:**78–86.

Elitzur S, Elhasid R, Ben-Barak A, Avrahami G, Blumenfeld Z. Long-term ovarian reserve and fertility outcomes in female survivors of childhood acute lymphoblastic leukemia. *Leukemia* 2021;**35:**2324–2331.

Evranos B, Faki S, Polat SB, Çakır B, Ersoy R, Çakır B. Effects of radioactive iodine therapy on ovarian reserve: a prospective pilot study. *Thyroid* 2018;**28:**1570–1578.

George SA, Williamson Lewis R, McKenzie L, Cherven B, Patterson BC, Effinger KE, Mertens AC, Meacham LR. Assessment of ovarian function in adolescents and young adults after childhood cancer treatment. *Pediatr Blood Cancer* 2019;**66:**e27981.

Gharwan H, Devata S, Griffiths EA, Wilding G, Wang ES, Wetzler M, Fetterly G, Sait S, Block AW, Starostik P et al. Female fertility following dose-adjusted EPOCH-R chemotherapy in primary mediastinal B-cell lymphomas. *Leuk Lymphoma* 2016;**57:**1390–1398.

Goldfarb SB, Turan V, Bedoschi G, Taylan E, Abdo N, Cigler T, Bang H, Patil S, Dickler MN, Oktay KH. Impact of adjuvant chemotherapy or tamoxifen alone on the ovarian reserve of young women with breast cancer. *Breast Cancer Res Treat* 2021;**185:**165–173.

Gracia CR, Sammel MD, Freeman E, Prewitt M, Carlson C, Ray A, Vance A, Ginsberg JP. Assessing the impact of cancer therapies on ovarian reserve. *Fertil Steril* 2012;**97:**134–140.

Gupta AA, Donen RM, Sung L, Vignola A, Grant R, Mitchell L, Koren G, Lee-Greenberg M, Barr R. Anti-Müllerian hormone in female adolescent cancer patients before, during, and after completion of therapy: a pilot feasibility study. *J Pediatr Hematol Oncol* 2016;**38:**355–359.

Hamre H, Kiserud CE, Ruud E, Stokke T, Fosså SD. Gonadal function and parenthood 20 years after treatment for childhood lymphoma: a cross-sectional study. *Pediatr Blood Cancer* 2012;**59:**271–277.

Hamy A-S, Porcher R, Esber N, Cuvier C, Giacchetti S, Coussy F, Hocini H, Tournant B, Perret F, Baulieu F et al. Anti-Müllerian hormone in breast cancer patients treated with chemotherapy: a retrospective evaluation of subsequent pregnancies. *Reprod Biomed Online* 2016;**32:**299–307.

Henry NL, Xia R, Schott AF, McConnell D, Banerjee M, Hayes DF. Prediction of postchemotherapy ovarian function using markers of ovarian reserve. *Oncologist* 2014;**19:**68–74.

Jin HY, Yeo Y, Kim DH, Chung NG, Cho B, Kim HK. Gonadal function in female adolescent and young adult survivors of childhood cancer. *J Adolesc Young Adult Oncol* 2023 (in press).

Jingrukwong L, Natesirinilkul R, Charoenkwan P, Sirikul W, Katanyuwong P, Chattipakorn N, Chattipakorn SC. Assessing the ovarian reserve in girls who survived childhood leukemia using anti-Müllerian hormone. *Pediatr Blood Cancer* 2025 (in press).

Kim HA, Lee JW, Nam SJ, Park W, Kil WH, Lee JE, Yi HW, Lee SK, Kim JH. Post-chemotherapy serum anti-Müllerian hormone level predicts ovarian function recovery. *Breast Cancer Res Treat* 2018;**171:**333–340.

Krawczuk-Rybak M, Leszczynska E, Chomanicka I, Zelazowska-Rutkowska B. The influence of different intensity of treatment on hormonal markers of gonadal function in acute lymphoblastic leukemia survivors. *J Pediatr Hematol Oncol* 2019;**41:**357–361.

Krawczuk-Rybak M, Leszczynska E, Poznanska M, Zelazowska-Rutkowska B, Wysocka J. Anti-Müllerian hormone as a sensitive marker of ovarian function in young cancer survivors. *Int J Cancer* 2013;**133:**2186–2192.

Krawczuk-Rybak M, Leszczynska E, Zelazowska-Rutkowska B, Wysocka J. The progressive reduction in the ovarian reserve in young women after anticancer treatment. *J Pediatr Hematol Oncol* 2013;**35:**e147–e151.

Lambertini M, Ceppi M, Poggio F, Peccatori FA, Azim HA, Ugolini D, Del Mastro L, Loibl S, Demeestere I, Minisini AM et al. Impact of taxanes, endocrine therapy, and deleterious germline BRCA mutations on anti-Müllerian hormone levels in early breast cancer patients treated with anthracycline- and cyclophosphamide-based chemotherapy. *Breast Cancer Res Treat* 2019;**175:**135–144.

Lambertini M, Havas J, Grinshpun A, Di Meglio A, Ignatiadis M, Desmedt C, Poggio F, Ceppi M, Del Mastro L, Blondeaux E et al. Predicting ovarian function loss after chemotherapy and anti-HER2 therapy in young breast cancer patients. *J Natl Cancer Inst* 2025;**117:**2317–2326.

Lee D-Y, Kim HJ, Kim SW, Cho CH, Kim MR, Lee SK, Kim TH, Kim JH. Prediction of ovarian function recovery in young breast cancer patients after protection with gonadotropin-releasing hormone agonist during chemotherapy. *Breast Cancer Res Treat* 2018;**168:**91–98.

Lee DY, Koo HJ, Yoon BK, Choi D. Prediction of successful ovarian protection using gonadotropin-releasing hormone agonists during chemotherapy in young estrogen receptor-negative breast cancer patients. *Breast Cancer Res Treat* 2020;**179:**47–53.

Leiper A, Houwing M, Davies EG, Rao K, Burns S, Morris E, Laven J, van der Kooi AL, van den Heuvel Eibrink M, Nussey S. Anti-Müllerian hormone and inhibin B after stem cell transplant in childhood: a comparison of myeloablative, reduced intensity and treosulfan-based chemotherapy regimens. *Bone Marrow Transplant* 2020;**55:**1985–1995.

Leonard RCF, Adamson DJA, Bertelli G, Mansi J, Donat CK, Mcgee P, Kerr GR, Anderson RA. GnRH agonist for protection against ovarian toxicity during chemotherapy for early breast cancer: the Anglo Celtic Group OPTION trial. *Ann Oncol* 2017;**28:**1811–1816.

Li X, Liu S, Ma L, Chen X, Weng H, Huang R, Yu Y, Zong X. Can anti-Müllerian hormone be a reliable biomarker for assessing ovarian function in women post-chemotherapy? *Cancer Manag Res* 2020;**12:**8171–8181.

Lie Fong S, Laven JSE, Hakvoort-Cammel FGAJ, Schipper I, Visser JA, Themmen APN, Drop SLS, van den Heuvel-Eibrink MM. Assessment of ovarian reserve in adult childhood cancer survivors using anti-Müllerian hormone. *Hum Reprod* 2009;**24:**982–990.

Loubersac S, Joueidi Y, Godbout A, Barranger E, Rosolowski M, Colomban O, You B, Lambertini M, Morice P, Salmon RJ, Freour T. Evolution of serum anti-Müllerian hormone level in young women treated with chemotherapy for breast cancer according to basal AMH level. *J Gynecol Obstet Hum Reprod* 2020;**49:**101702.

Lunsford AJ, Whelan K, McCormick K, McLaren JF. Anti-Müllerian hormone as a measure of reproductive function in female childhood cancer survivors. *Oncologist* 2014;**19:**183–189.

Lutchman Singh K, Muttukrishna S, Stein RC, McGarrigle HH, Patel A, Parikh B, Groome NP, Davies MC, Chatterjee R. Predictors of ovarian reserve in young women with breast cancer. *Br J Cancer* 2007;**96:**1808–1816.

Malisic E, Malisic D, Krtolica K. Assessment of ovarian function after chemotherapy in women with early and locally advanced breast cancer from Serbia. *Arch Gynecol Obstet* 2018;**297:**423–430.

Mittica M, Quaresmini D, Albrizio M, Cagnazzo C, Corcione S, Daniele L, Volpe G, Genta S, Scagliotti GV, Donadio M. Cross-sectional and prospective study on anti-Müllerian hormone changes in a cohort of pre-menopausal women with a history of differentiated thyroid cancer. *Front Endocrinol (Lausanne)* 2020;**11:**519.

Miyoshi Y, Ohta H, Namba N, Nakayama H, Ono K, Hosokawa S, Tachibana M, Inoue M, Yumura-Yagi K, Hara J et al. Low serum concentrations of anti-Müllerian hormone are common in 53 female childhood cancer survivors. *Horm Res Paediatr* 2012;**78:**232–236.

Morarji K, McArdle O, Hui K, Mote P, Beith J, Gebski V, Warrier SK, Guo L, Boyle FM. Ovarian function after chemotherapy in young breast cancer survivors. *Clin Oncol (R Coll Radiol)* 2017;**29:**456–463.

Mörse H, Elfving M, Lindgren AC, Baecklund F, Holmqvist AS, Lundell M, Perez-Vicente R, Ljungman G. Severe gonadotoxic insult manifests early in young girls treated for Ewing sarcoma. *Acta Oncol* 2016;**55:**1000–1005.

Nielsen SN, Andersen AN, Schmidt KT, Rechnitzer C, Schmiegelow K, Bentzen JG, Macklon KT. A 10-year follow up of reproductive function in women treated for childhood cancer. *Reprod Biomed Online* 2013;**27:**299–305.

Nies M, Cantineau AEP, Arts EGJM, Muller Kobold AC, van der Veen BS, Brouwers AH, Links TP, Dullaart RPF, van der Meer P, Fischbach RK et al. Long-term effects of radioiodine treatment on female fertility in survivors of childhood differentiated thyroid carcinoma. *Thyroid* 2020;**30:**547–554.

Nilsson S, Ekman T, Akesson K, Jarfelt M, Björk J, Dano H. A survey of ovarian reserve and quality of life in female survivors of pediatric cancer. *Acta Obstet Gynecol Scand* 2022;**101:**182–190.

Nitzschke M, Raddatz D, Strik A, Matthias W, Ramadori G. GnRH analogs do not protect ovaries from chemotherapy-induced ultrastructural injury in Hodgkin's lymphoma patients. *Arch Gynecol Obstet* 2010;**282:**83–88.

Nyström A, Khomyakova E, Mörse H, Elfving M, Lindgren AC, Holmqvist A, Perez-Vicente R, Lundell M, Ljungman G. Anti-müllerian hormone compared with other ovarian markers after childhood cancer treatment. *J Adolesc Young Adult Oncol* 2019;**8:**420–427.

Palinska-Rudzka KE, Ghobara T, Parsons N, Milner J, Lockwood G, Hartshorne GM. Five-year study assessing the clinical utility of anti-Müllerian hormone measurements in reproductive-age women with cancer. *Reprod Biomed Online* 2019;**39:**712–720.

Parissone F, Vero V, Sini I, Mancuso F, Gatto M, Lentini CM, Tafi E, Artuso I, Fozza A, Serafini A et al. Female adolescents and young women previously treated for pediatric malignancies: assessment of ovarian reserve and gonadotoxicity risk stratification for early identification of patients at increased infertility risk. *J Adolesc Young Adult Oncol* 2021;**10:**555–561.

Roshandel R, Anazodo A, Stern CJ, Agresta F, Sullivan E. Female reproductive function after treatment of childhood acute lymphoblastic leukemia. *J Adolesc Young Adult Oncol* 2021;**10:**258–268.

Shandley LM, Spencer JB, Fothergill A, Mertens AC, Manatunga A, Rashid A, Cottrell HN, Fridman M, Goodman DG, Milman LW et al. Impact of tamoxifen therapy on fertility in breast cancer survivors. *Fertil Steril* 2017;**107:**243–252.

Signorino C, Bencini E, Tonod A, Scalini P, Tamburini A, Casini T, Sardi I, Stagi S. Ovarian function in adolescents and young adults undergoing cancer treatment: biochemical and ultrasound marker analysis. *J Adolesc Young Adult Oncol* 2025 (in press).

Silva C, Caramelo O, Almeida-Santos T, Ribeiro Rama AC. Adverse reproductive health outcomes in a cohort of young women with breast cancer exposed to systemic treatments. *J Ovarian Res* 2019;**12:**102.

Singh MP, Bhatt S, Sinha V, Mastan A, Konar K, Saha D. Age and serum anti-Müllerian hormone levels as predictors of time to return of menses after chemotherapy. *Reprod Sci* 2025 (in press).

Su HI, Sammel MD, Green J, Velders L, Stankiewicz C, Matro J, Freeman EW, Gracia CR, DeMichele A. Antimullerian hormone and inhibin B are hormone measures of ovarian function in late reproductive-aged breast cancer survivors. *Cancer* 2010;**116:**592–599.

Su HI, Sammel MD, Gracia CR, Bharat S, Green J, Stankiewicz C, DeMichele A. Modeling variation in the reproductive lifespan of female adolescent and young adult cancer survivors using AMH. *J Clin Endocrinol Metab* 2020;**105:**2740–2751.

Thomas-Teinturier C, El Fayech C, Oberlin O, Pacquement H, Haddy N, Jackson A, Diallo I, de Vathaire F. Ovarian reserve after treatment with alkylating agents during childhood. *Hum Reprod* 2015;**30:**1437–1446.

Trapp E, Steidl J, Rack B, Schneeweiss A, Lichtenegger W, Beckmann MW, Fasching PA, Taran FA, Fehm TN, Simon W et al. Anti-Müllerian hormone (AMH) levels in premenopausal breast cancer patients treated with taxane-based adjuvant chemotherapy: a translational research project of the SUCCESS A study. *Breast* 2017;**35:**130–135.

van Beek RD, van den Heuvel-Eibrink MM, Laven JSE, de Jong FH, Themmen APN, Hakvoort-Cammel FGAJ, van den Bos C, van der Pal HJH, Pieters R, Drop SLS. Anti-Müllerian hormone is a sensitive serum marker for gonadal function in women treated for Hodgkin's lymphoma during childhood. *J Clin Endocrinol Metab* 2007;**92:**3869–3874.

van der Kooi AL, van den Heuvel-Eibrink MM, van Noortwijk A, Neggers SJCMM, Pluijm SMF, Laven JSE, Visser JA. Longitudinal follow-up in female childhood cancer survivors: no signs of accelerated ovarian function loss. *Hum Reprod* 2017;**32:**2475–2484.

van Velsen EFS, van Nederveen FH, van Kemenade FJ, Peeters RP, Visser WE. Longitudinal analysis of the effect of radioiodine therapy on ovarian reserve in females with differentiated thyroid cancer. *Thyroid* 2020;**30:**580–587.

Wenners A, Grambach J, Schulz J, Meinfelder M, Weimer JP, Mundhenke C, Jonat W, Maass N, Bauerschlag DO. Reduced ovarian reserve in young early breast cancer patients: preliminary data from a prospective cohort trial. *BMC Cancer* 2017;**17:**632.

Yaish I, Azem F, Gutfeld O, Silber Z, Porat S, Shahar L, Shefer G, Limor R, Stern N, Shafir L et al. A single radioactive iodine treatment has a deleterious effect on ovarian reserve in women with thyroid cancer: results of a prospective pilot study. *Thyroid* 2018;**28:**522–527.

Yu B, Douglas N, Ferin MJ, Nakhuda GS, Fratta L, Sauer MV, Lobo RA. Changes in markers of ovarian reserve and endocrine function in young women with breast cancer undergoing adjuvant chemotherapy. *Cancer* 2010;**116:**2099–2105.

Zhong Y, Lin Y, Cheng X, Zhou X, Shi Q, He W, Wang J, Zhou H, Hua X, Liu J. GnRHa for ovarian protection and the association between AMH and ovarian function during adjuvant chemotherapy for breast cancer. *J Cancer* 2019;**10:**4278–4285.
